# Validation and refinement of the revised 2017 European LeukemiaNet genetic risk stratification of acute myeloid leukemia

**DOI:** 10.1038/s41375-020-0806-0

**Published:** 2020-03-30

**Authors:** Tobias Herold, Maja Rothenberg-Thurley, Victoria V. Grunwald, Hanna Janke, Dennis Goerlich, Maria C. Sauerland, Nikola P. Konstandin, Annika Dufour, Stephanie Schneider, Michaela Neusser, Bianka Ksienzyk, Philipp A. Greif, Marion Subklewe, Andreas Faldum, Stefan K. Bohlander, Jan Braess, Bernhard Wörmann, Utz Krug, Wolfgang E. Berdel, Wolfgang Hiddemann, Karsten Spiekermann, Klaus H. Metzeler

**Affiliations:** 1 Laboratory for Leukemia Diagnostics, Department of Medicine III, University Hospital, LMU Munich, Munich, Germany; 2 German Cancer Consortium (DKTK), Partner Site Munich, Munich, Germany; 3 German Cancer Research Center (DKFZ), Heidelberg, Germany; 4 Research Unit Apoptosis in Hematopoietic Stem Cells, Helmholtz Zentrum München, German Research Center for Environmental Health (HMGU), Munich, Germany; 5 Institute of Biostatistics and Clinical Research, Westfälische Wilhelms-Universität Münster, Münster, Germany; 6 Department of Molecular Medicine and Pathology, University of Auckland, Auckland, New Zealand; 7 Department of Oncology and Hematology, Hospital Barmherzige Brüder, Regensburg, Germany; 8 Charité University Hospital Berlin, Berlin, Germany; 9 Hospital Leverkusen, Leverkusen, Germany; 10 Department of Medicine A, University Hospital Münster, Münster, Germany

**Keywords:** Acute myeloid leukaemia, Risk factors, Translational research, Genetics research, Cancer genetics

## Abstract

The revised 2017 European LeukemiaNet (ELN) recommendations for genetic risk stratification of acute myeloid leukemia have been widely adopted, but have not yet been validated in large cohorts of AML patients. We studied 1116 newly diagnosed AML patients (age range, 18–86 years) who had received induction chemotherapy. Among 771 patients not selected by genetics, the ELN-2017 classification re-assigned 26.5% of patients into a more favorable or, more commonly, a more adverse-risk group compared with the ELN-2010 recommendations. Forty percent of the cohort, and 51% of patients ≥60 years, were classified as adverse-risk by ELN-2017. In 599 patients <60 years, estimated 5-year overall survival (OS) was 64% for ELN-2017 favorable, 42% for intermediate-risk and 20% for adverse-risk patients. Among 517 patients aged ≥60 years, corresponding 5-year OS rates were 37, 16, and 6%. Patients with biallelic *CEBPA* mutations or inv(16) had particularly favorable outcomes, while patients with mutated *TP53* and a complex karyotype had especially poor prognosis. *DNMT3A* mutations associated with inferior OS within each ELN-2017 risk group. Our results validate the prognostic significance of the revised ELN-2017 risk classification in AML patients receiving induction chemotherapy across a broad age range. Further refinement of the ELN-2017 risk classification is possible.

## Introduction

In 2010, an international expert panel on behalf of the European LeukemiaNet (ELN) published guidelines for the diagnosis and management of acute myeloid leukemia (AML) in adults [1]. In this initial version of the ELN guidelines, a standardized reporting system for genetic alterations was proposed that integrated cytogenetic and molecular data to classify patients into four genetic groups. Subsequent studies showed this classification is prognostically relevant in intensively treated AML patients [2, 3]. The ELN-2010 genetic classification has subsequently found widespread adoption in clinical practice and in clinical trials.

In 2017, an updated version of the ELN recommendations has been published [4]. The ELN-2017 guidelines include an updated genetic risk stratification system incorporating additional cytogenetic and molecular prognostic factors. In the ELN-2017 risk stratification, the distinction between the Intermediate-I category (including only patients with normal cytogenetics) and the intermediate-II category (including patients with intermediate-risk abnormal karyotypes) has been eliminated, and consequently the number of risk categories has been reduced from four to three. Other major changes in the ELN-2017 classification include the addition of mutations in three genes (*ASXL1*, *RUNX1* and *TP53*) that are now considered adverse genetic markers, stratification of patients with *FLT3* internal tandem duplications (ITD) based on the ITD-to-wild-type allelic ratio, and the recognition that only biallelic, but not monoallelic, *CEBPA* mutations associate with favorable outcomes. In contrast to the ELN-2010 classification, the use of gene mutations for risk stratification is no longer restricted to patients with normal cytogenetics, reflecting the growing body of data on the prognostic relevance of gene mutations in AML.

While each individual cytogenetic and molecular marker included in the ELN-2017 classification has been repeatedly and convincingly shown to associate with patient outcomes, few studies so far have attempted to validate the proposed new classification on the whole [5, 6]. Importantly, existing validation studies were limited to younger AML patients, and one study excluded certain genetic subsets (*CEBPA*-mutated patients) [5]. Therefore, a comprehensive validation of the revised ELN-2017 classification in a large cohort of intensively treated younger and older AML patients is still lacking. We set out to test the prognostic relevance of the ELN-2017 risk groups in AML patients receiving induction chemotherapy on clinical trials, and to compare the revised risk categories to the ELN-2010 genetic groups. Our study provides a reference data set for future studies employing the ELN-2017 classification.

## Subjects and methods

### Patients and inclusion criteria

Our study included a total of 1116 previously untreated adult AML patients who had been enrolled on two subsequent multicenter phase III trials of the German AML Cooperative Group (AMLCG-1999, clinicaltrials.gov identifier NCT00266136, *n* = 857; and AMLCG-2008, NCT01382147, *n* = 259) [7–9]. Treatment regimens are summarized in the Supplementary Methods and Supplementary Fig. 1. AML was diagnosed according to World Health Organization (WHO) 2008 criteria [10]. The present analysis includes 771 patients selected solely based on the availability of cytogenetic data and a suitable bone marrow (BM) or peripheral blood (PB) specimen for mutation testing. An extension cohort of 345 patients with cytogenetically normal AML (CN-AML) treated on the AMLCG-1999 trial was included in the outcome analyses. These patients were not included in analyses of patient proportions assigned into the various ELN risk groups, or in analyses of patient reclassification between the ELN-2010 and ELN-2017 recommendations, to avoid bias due to an overrepresentation of CN-AML. All study protocols were in accordance with the Declaration of Helsinki and approved by the institutional review boards of participating centers. All patients provided written informed consent for inclusion on the clinical trial and genetic analyses.

### Genetic analyses, measurement of *FLT3*-ITD allelic ratio, and risk group assignment

Metaphase cytogenetics were analyzed centrally. For molecular analyses, mononuclear cells were enriched from pretreatment BM or PB by Ficoll density gradient centrifugation. Testing for *NPM1* and *CEBPA* gene mutations was performed from cDNA by polymerase chain reaction (PCR) followed by melting curve analysis or fragment analysis, respectively [11, 12]. *FLT3* ITD-to-wild-type allelic ratio was determined by PCR and fragment analysis from gDNA [13]. Mutations in 68 genes recurrently mutated in myeloid malignancies, including *NPM1*, *FLT3*, *CEBPA*, *ASXL1*, *RUNX1*, and *TP53*, were identified by targeted gDNA sequencing, with a limit of detection of 2% variant allele frequency [14, 15]. Ambiguities in ELN-2017 risk group assignment were resolved as described in the Supplement.

### Statistics

We studied associations between ELN genetic groups and other patient characteristics using Fisher’s exact test for categorical and the Wilcoxon rank-sum test for continuous variables. Analyses of treatment outcomes used commonly accepted definitions of complete remission (CR), relapse-free survival (RFS) and overall survival (OS) (Supplementary Methods) [4, 16]. For time-to-event analyses, we calculated survival estimates using the Kaplan–Meier method and compared groups by the log-rank test. We used multivariable logistic regression models to analyze factors associated with achievement of CR, and Cox proportional hazards models for survival endpoints. Statistical analyses were performed using R version 3.5.2 (R Foundation for Statistical Computing, Vienna, Austria).

## Results

### Association of the ELN-2017 categories with baseline demographics and comparison to the ELN-2010 genetic groups

**Fig. 1:**
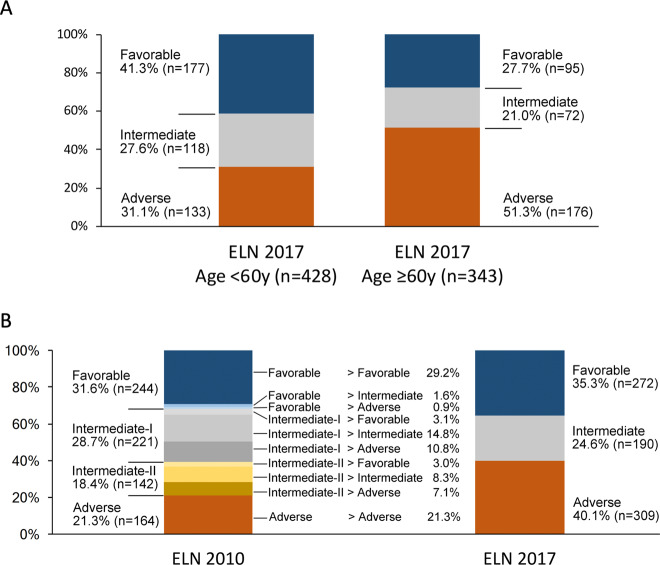
Distribution of ELN risk categories among intensively treated AML patients. **a** Distribution of the ELN-2017 risk categories in intensively treated AML patients aged <60 years (left) and in patients aged ≥60 years (right). **b** Comparison of risk group assignment according to the ELN-2010 and ELN-2017 genetic groups, and re-distribution of risk categories with the updated classification.

Among 771 newly diagnosed AML patients who were selected solely based on the availability of material for genetic analyses (median age, 57 years, range, 18–86 years), 272 (35%) were classified as favorable, 190 (25%) as intermediate, and 309 (40%) as adverse-risk according to the ELN-2017 recommendations (“baseline cohort”, Table 1). ELN-2017 adverse-risk patients were significantly older (median, 62 years) than intermediate- or favorable-risk patients (median, 53 and 54 years, respectively; *P* < 0.0001). Among patients <60 years of age, 41% were assigned to the favorable-risk group, 28% were intermediate risk, and 31% were adverse-risk. Among patients aged ≥60 years, only 28% were favorable-risk, 21% intermediate risk, while 51% belonged to the adverse-risk group (Fig. 1a). Of note, 47% of male patients had adverse-risk features, compared with only 33% of women (Table 1, Supplementary Fig. 2A; *P* = 0.0002). This difference was largely due to a lower prevalence of *NPM1* mutations and higher prevalence of *RUNX1* and *ASXL1* mutations among male patients (*P* < 0.0001, respectively). Adverse-risk patients also more frequently had secondary AML, had lower leukocyte counts (Supplementary Fig. 2B) and tended to have lower BM blast percentages compared with intermediate- or favorable-risk patients.

**Table 1:** Patient characteristics according to ELN-2017 risk group.

	ELN-2017 risk group			
Variable	Favorable	Intermediate	Adverse	*P*
Patient number	*n* = 272	*n* = 190	*n* = 309	
**Age** [years], median (range)	53 (18–86)	54 (18–77)	62 (21–80)	<0.0001
**Male sex**	113 (42%)	94 (49%)	181 (59%)	0.0002
**Disease type**				0.0006
*De novo* AML	248 (91%)	162 (85%)	244 (79%)	
Secondary AML	12 (4%)	17 (9%)	44 (14%)	
Therapy-related AML	12 (4%)	11 (6%)	21 (7%)	
**WBC** [×10^9^/L], median (range)	23.9 (0.9–316)	31.4 (0.6–486)	13.2 (0.5–406)	<0.0001
**Bone marrow blasts [%]**, median (range)	80 (6–100)	82 (10–100)	77 (9–100)	0.03
**FAB category**				<0.0001
M0	2	6	32	
M1	46	55	71	
M2	83	39	93	
M4	85	36	56	
M5	27	36	27	
M6	5	6	13	
M7	1	1	2	
Unknown	23	12	15	
**MRC cytogenetic risk category**				–
Favorable	81 (30%)	0	0	
Intermediate	189 (69%)	184 (97%)	157 (51%)	
Adverse	2 (1%)	6 (3%)	152 (49%)	
**ELN-2010 genetic group**				–
Favorable	225 (83%)	12 (6%)	7 (2%)	
Intermediate-I	24 (9%)	114 (60%)	83 (27%)	
Intermediate-II	23 (8%)	64 (34%)	55 (18%)	
Adverse	0	0	164 (53%)	
**Gene mutations detected in pretreatment sample**^**a**^				
* NPM1*	160 (59%)	76 (40%)	8 (3%)	<0.0001
* FLT3-*ITD	43 (16%)	92 (48%)	60 (19%)	<0.0001
- Low allelic ratio	41 (15%)	16 (8%)	20 (6%)	
- High allelic ratio	2 (1%)	76 (40%)	40 (13%)	
*CEBPA*	42 (15%)	16 (8%)	8 (3%)	<0.0001
- Mono-allelic	11 (4%)	16 (8%)	8 (3%)	
- Bi-allelic	31 (11%)	0	0	
*RUNX1*	3 (1%)	0	111 (36%)	<0.0001
*ASXL1*	11 (4%)	2 (1%)	77 (25%)	<0.0001
*TP53*	1 (<1%)	3 (2%)	71 (23%)	<0.0001
*DNMT3A*	90 (33%)	86 (46%)	66 (21%)	<0.0001
*TET2*	50 (18%)	33 (17%)	37 (12%)	0.07
*IDH1*	23 (8%)	13 (7%)	14 (5%)	0.15
*IDH2*	35 (13%)	26 (14%)	43 (14%)	0.93
*NRAS*	79 (29%)	27 (14%)	50 (16%)	0.0001
*KRAS*	19 (7%)	14 (7%)	15 (5%)	0.41
*WT1*	33 (12%)	34 (18%)	34 (11%)	0.08
*SRSF2*	14 (5%)	6 (3%)	55 (18%)	<0.0001
*PTPN11*	34 (13%)	8 (4%)	30 (10%)	0.007
*STAG2*	6 (2%)	14 (7%)	34 (11%)	0.0001
*BCOR*	3 (1%)	12 (6%)	41 (13%)	<0.0001
*RAD21*	23 (8%)	13 (7%)	7 (2%)	0.0020
*KMT2A* PTD	1 (<1%)	24 (13%)	31 (10%)	<0.0001
*KMT2A* PTD *status unknown*	20	4	6	

*WBC* white blood cell count, *FAB* French-American British classification, *MRC* British Medical Research Council, *ELN* European LeukemiaNet, *ITD* internal tandem duplication, *PTD* partial tandem duplication.

^a^Genes mutated in ≥5% of patients in the baseline cohort are listed.

Based on the previous ELN-2010 genetic groups, 244 of the 771 patients (32%) were classified as favorable, 221 (29%) as intermediate-I, 142 (18%) as intermediate-II and 164 (21%) as adverse (Fig. 1b). In the ELN-2017 risk stratification system, the distinction between the intermediate-I and intermediate–II groups has been eliminated and the number of categories reduced from four to three, complicating side-by-side comparisons between the old and revised classification. However, when the ELN-2010 intermediate-I and intermediate-II groups were treated as one, the novel classification resulted in reclassification of 204 patients (26.5%) into a higher or lower prognostic category (Supplementary Table 1). The net effects were a 3.7 percentage point increase in the proportion of patients classified as favorable, and an 18.8 percentage point increase in the proportion of patients classified as adverse, while the proportion of patients classified into the intermediate-risk category decreased by 22.5 percentage points.

### Outcomes of AML patients classified according to the 2017 ELN risk stratification system

**Fig. 2:**
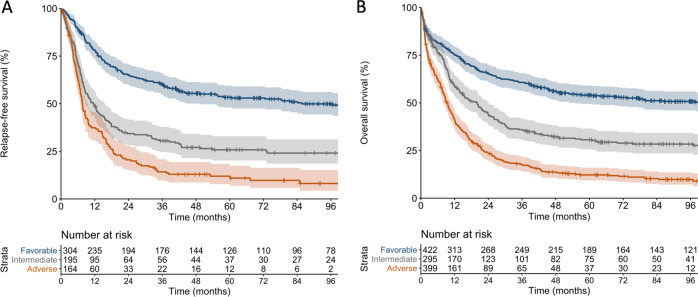
Outcomes of patients according to the ELN-2017 genetic risk groups. **a** Relapse-free survival and **b** overall survival according to the ELN-2017 categories in the entire cohort of 1116 patients (age range, 18–86 years).

**Fig. 3:**
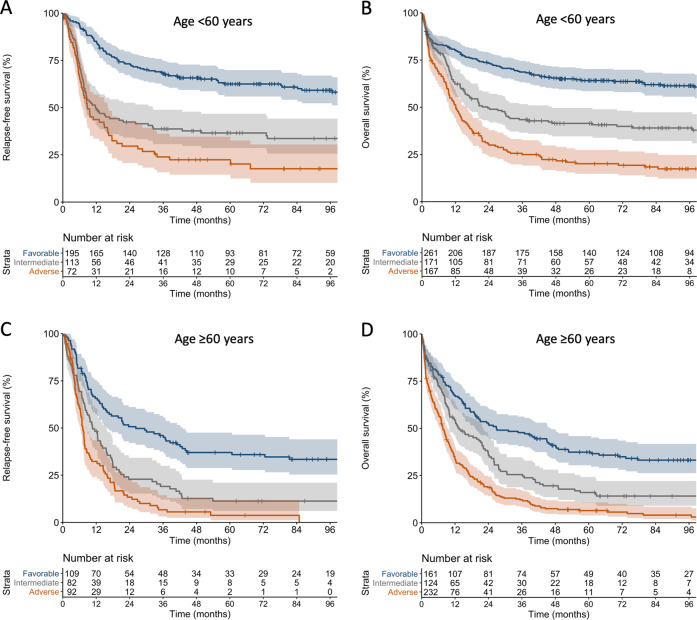
Outcomes of patients according to the ELN-2017 genetic risk groups, stratified by age group. **a** Relapse-free survival and **b** overall survival according to ELN-2017 categories in 599 patients aged <60 years. **c** Relapse-free survival and **d** overall survival according to ELN-2017 categories in 517 patients aged ≥60 years.

Outcome analyses included the previously described “baseline cohort” of 771 patients and an extension cohort of 345 patients with CN-AML, a subset particularly affected by the changes in the ELN-2017 classification, for a total of 1116 patients (“outcomes cohort”, Supplementary Table 2). All patients had received cytarabine plus adriamycin or mitoxantrone-based induction chemotherapy on phase III clinical trials, and none of the *FLT3*-mutated patients had received a tyrosine kinase inhibitor upfront. The median follow-up for survivors was 98 months [17]. According to the ELN-2017 recommendations, 422 patients in the outcomes cohort (38%) were classified as favorable, 295 (26%) as intermediate, and 399 (36%) as adverse-risk. Patients in the favorable- and intermediate-risk categories had similar remission rates, while adverse-risk patients had significantly lower CR rates (Table 2). Patients in the favorable, intermediate and adverse-risk categories showed progressively worse RFS and OS (Table 2, Fig. 2). These associations were also observed in the subgroups of patients aged <60 years (n = 599) or ≥60 years (*n* = 517) (Table 2, Fig. 3). Even though the ELN-2017 classification almost doubled the proportion of patients classified as adverse-risk compared with the ELN-2010 recommendations, 5-year OS of these patients was only 12% in the entire cohort and 6% in older patients.

**Table 2:** Outcomes according to the ELN-2017 genetic risk groups.

ELN-2017 genetic risk group	Complete remission		RFS		OS	
	*n* [%]	*P*	5-year RFS, % (95% CI)	*P*	5-year OS, % (95% CI)	*P*
**All patients (*n*** = **1116)**						
Favorable (*n* = 422)	305 (72)	<0.0001	53.4 (48.0–59.4)	<0.0001	54.0 (49.4–59.1)	<0.0001
Intermediate (*n* = 295)	195 (66)		25.8 (20.2–32.9)		30.6 (25.7–36.5)	
Adverse (*n* = 399)	164 (41)		11.9 (7.8–18.4)		12.2 (9.3–16.0)	
**Age** < **60 years (*n*** = **599)**						
Favorable (*n* = 261)	196 (75)	<0.0001	62.5 (55.9–69.8)	<0.0001	64.2 (58.5–70.3)	<0.0001
Intermediate (*n* = 171)	113 (66)		36.6 (28.6–46.8)		41.5 (34.6–49.7)	
Adverse (*n* = 167)	72 (43)		22.4 (14.5–34.6)		20.1 (14.7–27.5)	
**Age** ≥ **60 years (*n*** = **517)**						
Favorable (*n* = 161)	109 (68)	<0.0001	37.0 (28.8–47.5)	<0.0001	37.4 (30.4–45.9)	<0.0001
Intermediate (*n* = 124)	82 (66)		11.3 (6.1–21.0)		16.0 (10.6–24.2)	
Adverse (*n* = 232)	92 (40)		3.7 (1.2–11.9)		6.4 (3.9–10.7)	

*ELN* European LeukemiaNet, *RFS* relapse-free survival, *OS* overall survival, *CI* confidence interval.

**Fig. 4:**
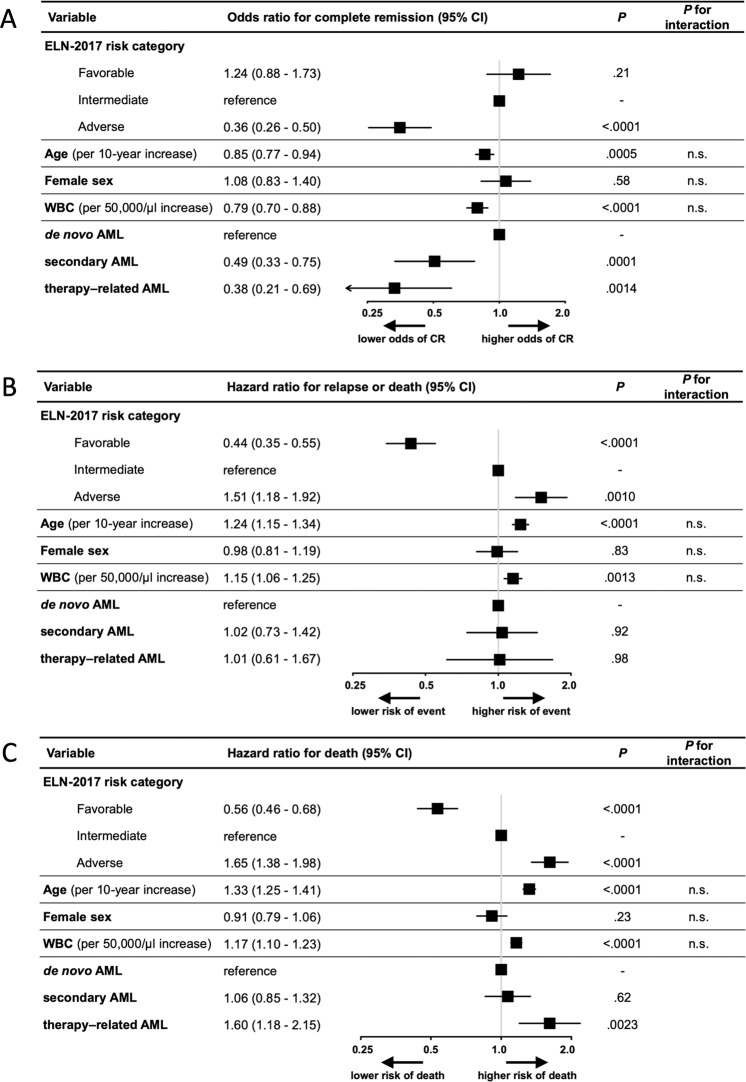
Multivariable analyses of outcomes according to the ELN-2017 genetic risk groups and other pretreatment prognostic variables. **a** Forrest plot showing odds ratios from a logistic regression model for achievement of complete remission. **b** Forrest plot showing hazard ratios from a Cox proportional hazards model for relapse-free survival. **c** Forrest plot showing hazard ratios from a Cox proportional hazards model for overall survival. Interaction *P* values refer to an interaction between the ELN-2017 risk groups and the respective variable. All multivariable models were stratified according to trial and induction therapy arm to account for potential differences in baseline risk between trials.

Since the ELN-2017 genetic risk groups show strong associations with other baseline variables including patient age, sex, leukocyte counts, and clinically defined secondary or treatment related AML (sAML/tAML) (Table 1), we constructed multivariable models adjusting for these factors. In a model for CR, ELN-2017 favorable- and intermediate-risk patients had similar odds of reaching remission, while adverse-risk patients had a significantly lower CR rate (Fig. 4a). Older age, higher leukocyte counts, and a clinical diagnosis of sAML/tAML associated with lower CR rates after adjustment for the ELN-2017 genetic categories. In a model for RFS, the risk of relapse or death for ELN-2017 favorable-risk patients was less than half compared with the intermediate-risk category, and adverse-risk patients had an ~1.5-fold higher risk (Fig. 4b). Older age and higher leukocyte counts were also linked to shorter RFS, while sAML or tAML were not associated with RFS after adjusting for the other factors. Regarding OS, favorable-risk patients had an ~50% reduced, and adverse-risk patients a 60% increased risk of death relative to the intermediate-risk group (Fig. 4c). Other factors associated with shorter survival were older age, higher leukocyte counts, and a diagnosis of tAML, but not sAML.

**Fig. 5:**
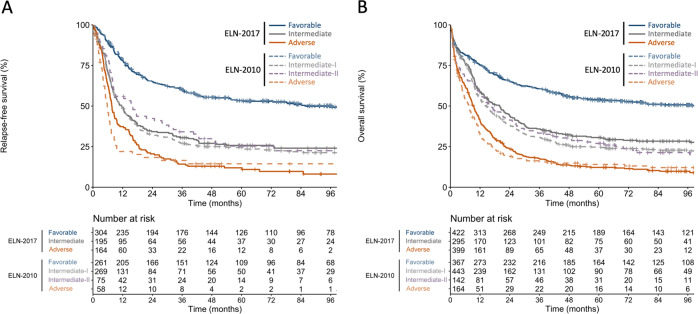
Outcomes of risk categories defined by ELN-2017 guidelines (solid lines) in comparison to the ELN-2010 risk categories (dashed lines). **a** Relapse-free survival and **b** overall survival.

The ELN-2017 prognostic classification resulted in better overall discrimination of risk groups compared with the ELN-2010 genetic groups, as shown by higher time-dependent areas under receiver-operating-characteristic curves for RFS and OS (Supplementary Fig. 3) [18]. This increase in prognostic value was due to the larger fractions of patients identified as favorable- or adverse-risk by the ELN-2017 system, while RFS and OS of the ELN-2017 favorable, intermediate and adverse-risk groups remained very similar to the ELN-2010 favorable, intermediate-I/II and adverse-risk categories (Fig. 5). Detailed outcomes analyses for patients re-classified into a lower-risk or higher-risk category in the ELN-2017 classification compared with the 2010 guidelines are provided in Supplementary Fig. 4.

### Postremission therapy

Overall, 664 patients in our cohort reached CR after protocol-specified induction therapy. Among those, 109 underwent allogeneic stem cell transplantation (alloSCT) and 46 underwent autologous transplantation (autoSCT) while in first CR (CR1), and 509 received only chemotherapy as postremission therapy (Supplementary Fig. 1). AlloSCT and autoSCT were performed in 25 and 12% of CR1 patients aged <60 years, respectively, compared with only 4 and <1% of those aged ≥60 years (Supplementary Fig. 5A). Therefore, analyses of outcomes according to postremission treatment received were restricted to CR1 patients younger than 60. Clinical characteristics of this subgroup (*n* = 381) are shown in Supplementary Table 3. Although most patients in our analysis were treated before the widespread use of molecular genetic risk factors for postremission therapy selection, there was a trend towards more frequent use of alloSCT among ELN-2017 adverse-risk compared with intermediate-risk or favorable-risk patients (36% vs. 27% vs. 20%; *P* = 0.06; Supplementary Fig. 5B).

Supplementary Figs. 6–8 depict RFS and OS of ELN-2017 favorable, intermediate and adverse-risk patients according to postremission therapy. In multivariable analyses adjusting for age, within each ELN-2017 risk group patients receiving alloSCT in CR1 had longer RFS compared with those receiving chemotherapy. However, alloSCT associated with improved OS only in the adverse-risk group (*P* = 0.05). Intermediate-risk patients receiving alloSCT in CR1 showed a nonsignificant trend towards improved OS, while in the favorable-risk group, OS was similar for patients receiving alloSCT or chemotherapy only. Of note, since postremission alloSCT assignment was not randomized, other factors besides genetic risk may have affected therapeutic decisions and thus biased these results.

### Outcomes of patients within genetic subsets of the ELN-2017 categories

Outcomes of specific genetic subsets within the ELN-2017 risk categories are presented in detail in the Supplementary Results, Supplementary Table 4, and Supplementary Figs. 9–14. Overall, these analyses support the changes introduced in the ELN-2017 guidelines, including the revised risk stratification based on *FLT3*-ITD allelic ratio and *NPM1* mutation status as well as the inclusion of *ASXL1* and *RUNX1* mutations as unfavorable markers. Within the ELN-2017 favorable-risk group, patients with inv(16)/t(16;16) or biallelic CEBPA mutations had superior OS, with an estimated 5-year OS of 70% respectively, compared with the other genetic subsets within this category which achieved 5-year survival rates between 48 and 51% (*P* = 0.0005, Supplementary Figs. 9A, B). On the other hand, within the ELN-2017 adverse-risk category, patients with complex karyotypes together with mutated *TP53* had particularly unfavorable outcomes with a 5-year RFS and OS of 0% (Supplementary Fig. 9E, F).

### Proposed refinement of the ELN-2017 prognostic stratification system

**Fig. 6:**
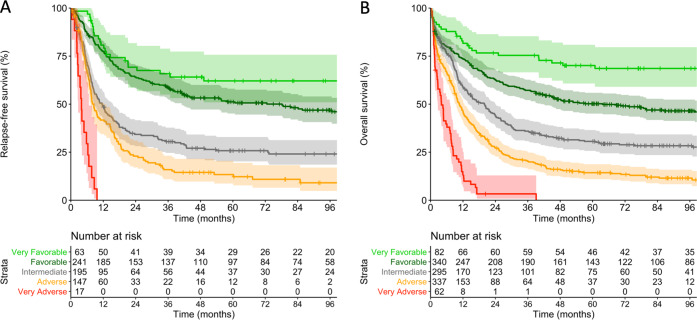
Outcomes of patients according to the proposed refinement of the ELN-2017 genetic risk groups. **a** Relapse-free survival and **b** overall survival in the entire cohort of 1116 patients (age range, 18–86 years).

Based on our analyses of genetic subsets within the ELN-defined risk groups and previously published data [19–23], we propose to further refine the ELN-2017 classification without introducing additional markers, by separating a “very favorable” subgroup (patients with inv(16)/t(16;16) or biallelic *CEBPA* mutations; *n* = 82) from the favorable category, and a “very adverse” subgroup (patients with *TP53* mutations and a complex karyotype; *n* = 62) from the adverse category. Using this refined classification, CR rates for the very favorable, favorable, intermediate, adverse and very adverse groups were 77, 71, 66, 44 and 27%, respectively (Supplementary Table 5). RFS and OS for the refined ELN-2017 classification are shown in Fig. 6 and Supplementary Fig. 15. Estimated OS rates at 5 years were 70% for very favorable, 50% for favorable, 31% for intermediate, 14% for adverse and 0% for very adverse patients. In multivariable analyses adjusting for potential confounders (Supplementary Fig. 16), the very adverse group of the refined classification had inferior CR rate, RFS and OS compared with the adverse group. The very favorable-risk subgroup had longer OS compared with the favorable subgroup, although CR rate and RFS were not significantly different. This OS difference was driven by survival after relapse (Supplementary Fig. 17), which was significantly longer for the very favorable compared with the favorable (*P* = 0.018) and to all other subgroups (*P* = 0.005), consistent with reports that patients with biCEBPA mutations or inv(16) are particularly responsive to salvage therapy [20, 24]. The proposed refinement of the ELN-2017 risk groups was successfully validated in an independent cohort of mostly younger AML patients treated on clinical trials of the AML-SG study group [19] (*n* = 1540; 83% aged <60 years; Supplementary Methods and Supplementary Fig. 18). We also tested whether the inclusion of additional gene mutations can further refine the ELN-2017 risk groups, and found that within each risk category mutated *DNMT3A* identified a subgroup with significantly inferior OS compared with *DNMT3A* wild-type patients (Supplementary Results and Supplementary Figs. 19 and 20).

## Discussion

Since the ELN recommendations for AML risk stratification by genetics were revised in 2017, the updated classification has been widely adopted. For example, the ELN-2017 risk stratification has been incorporated into the U.S. National Comprehensive Cancer Center Network (NCCN) clinical practice guidelines for AML [25]. Nevertheless, it is important to remember the ELN classification is an expert consensus statement, based on a synthesis of retrospective analyses from different cooperative groups, and mostly involving patients who had received intensive chemotherapy on clinical trials. While the individual prognostic markers recognized in the ELN-2017 recommendations are supported by retrospective studies, the entire risk stratification system was not validated in the primary publication [4]. Our retrospective analysis confirms that the ELN-2017 classification allows robust risk stratification of AML patients receiving cytarabine plus anthracycline/anthracenedione-based induction therapy. Compared with the ELN-2010 reporting system, ELN-2017 improves risk stratification by classifying more patients as favorable- or adverse-risk groups, and thus leaving fewer patients in the intermediate risk group. Thereby, the ELN-2017 classification facilitates discussions with patients about their individual prognosis at the time of initial diagnosis. We demonstrate the ELN-2017 classification is applicable in younger (<60 years) as well as in older (≥60 years) patients receiving induction chemotherapy. Of note, the prognostic relevance of the ELN-2017 categories is less clear in very old patients (≥75 y) who still undergo intensive treatment [26]. We observed that women were more likely to belong to ELN favorable group, while men more often fell into the adverse group due to a higher incidence of *NPM1* mutations and a lower incidence of *ASXL1* and *RUNX1* mutations in females, as described previously [14, 27–29]. Population-based analyses from the U.S. Surveillance, Epidemiology, and End Result (SEER) database also show male AML patients have worse OS [30], although analyses in other countries did not identify sex-specific survival differences [31].

Previously published studies suggest that the ELN-2017 prognostic groups can be further refined without including additional genetic markers. For example, leukemias with the core binding factor gene rearrangements, t(8;21) or inv(16)/t(16;16), both have relatively favorable outcomes. However, data from the CALGB and AML-SG study groups and the SEER registry have suggested that OS of patients with inv(16) is superior compared to those with t(8;21) [19–21], although this difference was not observed in other large cohorts [32]. Likewise, biallelic *CEBPA* mutations seem to delineate a patient subgroup with particularly good outcomes even compared with other favorable-risk groups [19, 24]. Since our findings are in line with these previous analyses, we suggest that the inv(16) and biallelic *CEBPA*-mutated subgroups should be regarded as prognostically “very favorable” with an expected 5-year OS of close to 80% in younger and 50% in older patients. On the other side of the spectrum, complex chromosomal alterations associate with poor outcomes. Within this group, “typical complex karyotypes” (i.e., those with deletions affecting chromosome arms 5q, 7q, and/or 17p) frequently co-exist with *TP53* mutations, and those patients have particularly dismal outcomes [22, 23]. In our cohort, none of the patients with *TP53* mutation and a complex karyotype achieved long-term survival. Thus, the available data consistently show that this genotype defines a “very unfavorable” subgroup of AML.

Since current treatment guidelines suggest allogeneic stem cell transplantation (alloSCT) as the preferred postremission treatment in suitable patients with adverse genetic risk [4, 25], the updated ELN classification would be expected to lead to an increase in the proportion of AML patients considered for alloSCT in first remission. Due to the enrollment period of the trials analyzed here, and since many patients now assigned to the ELN-2017 adverse-risk group were originally not classified as poor risk, <20% of ELN-2017 adverse patients received an alloSCT while in first remission. It is currently unclear if the increased proportion of adverse-risk patients recommended to undergo alloSCT will ultimately improve outcomes in this patient population. Despite this uncertainty, the ELN-2017 adverse-risk definition allows the identification of a large high-risk subgroup of AML patients who clearly have suboptimal outcomes with conventional induction chemotherapy. In particular, patients in the “very unfavorable” subgroup proposed here almost never achieve durable remissions when treated with induction chemotherapy, even if it is followed by allogeneic transplantation. We suggest these patients should not be offered conventional intensive chemotherapy and should be enrolled in clinical trials whenever possible.

In this context, our data not only validate the use of the ELN-2017 classification for pretreatment risk stratification in intensively treated AML patients, but can also serve as a benchmark for clinical trials evaluating novel therapeutic strategies. It is important to keep in mind that risk classification systems must always be interpreted in conjunction with treatment regimens, which may change over time. The studies that led to the ELN-2017 recommendations, as well as our validation analysis, included AML patients receiving traditional cytarabine-based induction chemotherapy. The addition of targeted agents, such as *FLT3* inhibitors or inhibitors of mutated *IDH1* or *IDH2* [33, 34], to standard induction therapy may alter the prognosis of patients receiving these novel agents and thus prompt changes in genetic risk classification. It is also uncertain if the ELN-2017 risk groups are applicable in patients treated with alternative first-line approaches such as hypomethylating agents [35, 36], venetoclax-based regimens [37, 38], novel targeted agents or immunotherapeutical approaches [39, 40].

Finally, predictions of long-term treatment outcomes based on pretreatment genetic characterization alone are far from perfect (Supplementary Fig. 3). Age, comorbidities, performance status and other important risk factors are not reflected in the ELN categories [41]. Moreover, analyses of measurable residual disease (MRD) during and after treatment by flow cytometry, quantitative PCR or next-generation sequencing have emerged as novel tools to assess response to therapy and prognosis [42–45]. One major current challenge thus is the development of algorithms that integrate pretreatment risk factors and longitudinal MRD measurements to guide individualized AML treatment. However, only prognostic factors that can be determined at the time of diagnosis can be used to guide selection of initial therapy. Our data demonstrate that pretreatment genetic risk stratification according to the ELN-2017 criteria identifies patient subgroups with a high chance of cure, as well as subgroups of patients who do not benefit from induction chemotherapy. Therefore, pretreatment genetic risk stratification will likely remain an integral part of AML treatment algorithms.

In summary, our study provides the first independent validation of the ELN-2017 recommendations for risk stratification by genetics in a large cohort of patients across a broad age range who were treated with induction chemotherapy on clinical trials. Compared with the previous ELN-2010 guidelines, the ELN-2017 recommendations result in more patients being classified as favorable risk, and significantly more patients classified as adverse-risk, and thereby improve overall risk assessment. Further refinement of the ELN-2017 classification is possible using variables already considered in the current guidelines, or by including additional genetic markers.

## Supplementary information

Supplemental Appendix
