# Supplementary material for: Validation and refinement of the revised 2017 European LeukemiaNet genetic risk stratification of acute myeloid leukemia
Source: Leukemia. 2020 Mar 30;34(12):3161–72. doi: 10.1038/s41375-020-0806-0 (PMC7685975; doi:10.1038/s41375-020-0806-0)
Supplement: Supplementary file 1 — Supplemental Appendix [file 41375_2020_806_MOESM1_ESM.pdf]

SUPPLEMENTAL APPENDIX TO

HEROLD ET AL.:

VALIDATION AND REFINEMENT OF THE REVISED 2017 EUROPEAN LEUKEMIANET GENETIC RISK STRATIFICATION OF ACUTE MYELOID LEUKEMIA

SUPPLEMENTAL INFORMATION ON PATIENTS AND METHODS

Treatment protocols

AMLCG-1999 trial

Induction therapy: In the AMLCG-1999 trial[1] (clinicaltrials.gov identifier NCT00266136; recruitment period, 1999 to 2011; patients included in this analysis were recruited between 1999 and 2004), patients <60 years were randomized to receive double induction with either one cycle of TAD-9 followed by one cycle of HAM on day 21 (TAD-HAM), or two cycles of HAM 21 days apart (HAM-HAM; TAD-9: thioguanine 100 mg/m2 twice daily on days 3-9, cytarabine 100 mg/m²/d continuous infusion on days 1+2 and 100 mg/m² twice daily on days 3-8, and daunorubicin 60 mg/m² on days 3-5; HAM: cytarabine 3 g/m² twice daily on days 1-3 and mitoxantrone 10 mg/m² on days 3-5). From 2002, 10% of patients aged ≤60 years (n=14 patients in this analysis) were randomized to a common standard arm of the German AML intergroup and received 7+3 induction therapy (cytarabine 100 mg/m²/d continuous infusion on days 1-7 and daunorubicin 60 mg/m² on days 3-5).[2]

Postremission treatment: Patients <60 years underwent upfront randomization to undergo consolidation either with one cycle of TAD-9 followed by three years of monthly cytarabine-based maintenance chemotherapy, or a single TAD-9 consolidation course followed by autologous stem cell transplantation (autoSCT) and no maintenance. Per the study protocol, all patients aged <60 years with HLA-matched sibling donors were to be offered allogeneic stem cell transplantation (alloSCT) in first CR, irrespective of cytogenetic or molecular risk factors. Patients ≥60 years were randomized to receive induction therapy with one cycle of either TAD-9 or HAM, followed by a second HAM cycle on day 21 only if ≥5% residual blasts were present in the BM on day 16. All patients ≥60 years were to receive one cycle of TAD-9 consolidation followed by maintenance therapy.

AMLCG-2008 trial

In the AMLCG-2008 trial[3] (NCT01382147; recruitment period, 2009 to 2012), patients <60 years and fit patients up to the age of 70 ('younger' patients) were randomized to receive either double induction chemotherapy with TAD-9 and HAM (21 days apart), or dose-dense induction therapy according to the sHAM regimen (cytarabine 3 g/m² [1 g/m² in patients ≥60 years] twice daily on days 1,2,8 and 9; and mitoxantrone 10 mg/m² on days 3,4,10 and 11). AlloSCT from an HLA-matched related or unrelated donor was the recommended postremission therapy for all younger patients achieving CR except those with favorable genetic features (defined as favorable cytogenetics or cytogenetically normal patients with mutated *NPM1* and no *FLT3*-ITD) and good response to induction chemotherapy (<10% blasts in a bone marrow aspirate obtained on d16 after start of induction therapy). For younger patients without a donor, those unable or unwilling to undergo allotransplantation, and those with a favorable risk profile, postremission therapy consisted of one cycle of TAD-9 for consolidation, followed by 3 years of cytarabine-based maintenance therapy.

Less fit patients aged ≥60 years, and all patients aged ≥70 years, were randomized to receive induction therapy according to the HAM regimen (cytarabine, 1g/m² per dose) followed by a second HAM induction cycle on day 21 only if a bone marrow aspirate on day 16 showed ≥5% blasts, or to dose-dense induction with sHAM (cytarabine, 1g/m² per dose). Postremission therapy in this group consisted of one cycle of TAD-9 for consolidation, followed by 3 years of maintenance therapy.

Definition of clinical end points

Clinical endpoints were defined, in accordance with generally accepted criteria,[4,5] as follows: Complete remission (CR) required a bone marrow (BM) aspirate with cellularity greater than 20% and maturation of all cell lines, less than 5% blasts and no Auer rods; and in the peripheral blood, an absolute neutrophil count of ≥1,500/µL, platelet count of ≥100,000/µL, and no leukemic blasts; and no evidence of extramedullary leukemia. Relapse was defined by the

presence of ≥5% BM blasts, or circulating leukemic blasts, or the development of extramedullary leukemia. Relapse-free survival (RFS) was measured from the date of CR until the date of relapse or death; patients alive and in CR were censored at last follow-up. Overall survival (OS) was measured from the date of study entry until the date of death, and patients alive at last follow-up were censored.

Ambiguities in ELN-2017 risk group assignment

We identified a small number of patients (approximately 1%) in which the ELN-2017 recommendations for genetic risk stratification did not allow an unambiguous risk group assignment. Specifically, four patients had mutated *NPM1* in the context of complex karyotypes, and were classified as adverse risk in agreement with recently published data.[6] Of these four patients, three achieved a CR. Two had alloSCT in first remission and were alive and relapse-free 3.4 and 8.2 years from diagnosis, the third patient received an alloSCT for early relapse and died 9 months after initial presentation. Subsequent therapy for the patient not achieving CR is unknown, but he was alive 2 years after initial diagnosis. One patient had a *FLT3*-ITD with a high allelic ratio and biallelic *CEBPA* mutations, and another patient had a *FLT3*-ITD with a high allelic ratio and an inversion inv(16). Both were assigned to the favorable-risk category, and were alive in CR1 approximately 14.5 years and 8.5 years after AML diagnosis, respectively. Four patients had mutated *TP53* in the presence of a balanced chromosomal translocation [inv(16), n=1 and t(9;11), n=3] and were assigned to the favorable- and intermediate-risk categories, respectively. The patient with mutated *TP53* and inv(16) received an allogeneic transplant in CR1 and was alive in remission 4 years after his initial diagnosis. Among the three patients with mutated *TP53* and t(9;11), one received alloSCT in CR1 but relapsed and died 2.5 years from AML diagnosis, while two did not achieve CR and died within the first two months from diagnosis.

Validation of the proposed refinement of the ELN-2017 classification in an independent patient cohort

To validate our proposed refinement of the ELN-2017 risk classification, we analyzed published genetic and survival data of patients treated on three consecutive clinical trials of the German AML-SG study group (n=1540).[7] In brief, the AML-HD98A trial included younger patients (18-60 years) who received induction chemotherapy with idarubicin, cytarabine and etoposide (ICE). Allogenic transplantation was offered to patients with adverse cytogenetic risk, intermediate-risk patients received allogeneic transplantation or intensive postremission chemotherapy, and low-risk patients received chemotherapy only. The AML-SG 07-04 trial included younger patients who were randomized to induction with either ICE or ICE plus all-trans retinoic acid (ATRA). In the AML-HD98B trial, patients aged ≥60 years were randomized to receive induction with either ICE or ICE plus ATRA.

For this cohort, information on *FLT3*-ITD-to-wild type allelic ratio is not publicly available. Patients that could not be reliably classified according to the ELN-2017 criteria, mostly due to missing data on *FLT3* allelic ratio, as well as patients with acute promyelocytic leukemia, were excluded, leaving 1192 patients for the validation cohort (median age, 51 years; range, 18-84 years; 83% aged <60 years). Of note, the lack of data on *FLT3*-ITD allelic ratio does not interfere with classifying patients into the proposed “very favorable” and “very adverse” subgroups, allowing us to use this cohort for the purpose of validating our refinement of the ELN-2017 classification.

SUPPLEMENTAL RESULTS

Outcomes of patients within genetic subsets of the ELN-2017 categories

We examined outcomes of specific genetic subsets with the ELN-2017 risk categories (Supplemental Table 4). Off note, since several of the resulting subgroups are relatively small and since these analyses were not adjusted for multiple testing, these results should be considered exploratory until further validation.

Within the ELN-2017 favorable risk group (Supplemental Figure 9A,B), patients with inv(16)/t(16;16) or biallelic CEBPA mutations had superior OS, with an estimated 5-year OS of 70% respectively, compared to the other genetic subsets within this category which achieved 5 year survival rates between 48% and 51% (*P*=.0005). Patients with mutated *NPM1* and a *FLT3*-ITD mutation with low allelic ratio (*FLT3*-ITD^{low}) had similar long-term outcomes compared to those with mutated *NPM1* and no *FLT3*-ITD, supporting the inclusion of the former subgroup in the favorable-risk category.

Within the ELN-2017 intermediate risk group (Supplemental Figure 9C,D), patients with t(9;11) and those with mutated *NPM1* and *FLT3*-ITD with a high allelic ratio (*FLT3*-ITD^{high}) had shorter median survival OS (11.2 months and 10.4 months, respectively) compared to patients with wild type *NPM1* and *FLT3*-ITD^{low}, and those grouped into the intermediate category due to absence of a risk-defining genotype (median OS, 27.1 and 26.1 months, respectively; *P*=.02). At 5 years from initial diagnosis, however, all subsets within the ELN-2017 intermediate-risk category had estimated OS rates between 20% and 40%.

Notably, within the ELN-2017 adverse-risk category (Supplemental Figure 9E,F), patients with complex karyotypes together with mutated *TP53* were the only subgroup with a 5-year RFS and OS of 0%. On the other hand, patients with non-t(9;11) *KMT2A* rearrangements had relatively favorable OS, yet this subgroup was relatively small. The remaining ELN-2017 adverse-risk subsets, including patients with complex karyotype but no *TP53* mutation, had 5-

year survival rates between approximately 10% and 20%. Mutations in *RUNX1* and *ASXL1* were newly recognized as adverse-risk markers in the ELN-2017 classification, and co-occurrence of these mutations was common (*P*<.001 for the association between both mutations). Outcomes were similar for patients with mutations in *ASXL1* only, *RUNX1* only, or mutations in both genes (Supplemental Figure 10), supporting the inclusion of both genes in the ELN-2017 system. The incorporation of the *FLT3*-ITD mutant-to-wild type allelic ratio represents another major change from the ELN-2010 guidelines. When we evaluated patient outcomes according to *FLT3*-ITD status and allelic ratio and *NPM1* mutation status, and without taking other genetic markers into account (Supplemental Figure 11), patients with mutated *NPM1* and *FLT3*-ITD^{low} had favorable long-term outcomes comparable to those with mutated *NPM1* and no *FLT3*-ITD. Of note, relapses occurred earlier in the *NPM1*^{mut}/*FLT3*-ITD^{low} group than in the *NPM1*^{mut}/*FLT3*-ITD^{neg} patients, yet RFS and OS at 5 years were similar. Patients with wild type *NPM1* and *FLT3*-ITD^{high} had inferior OS, while the remaining groups (*NPM1*^{mut}/*FLT3*-ITD^{high}, *NPM1*$^{wild\ type}$/ *FLT3*-ITD^{low} and *NPM1*^{wildtype}/FLT3-ITD^{neg}) all had similar OS rates at 5 years. These results support the risk stratification based on *NPM1* status and *FLT3*-ITD allelic ratio as introduced in the ELN-2017 guidelines.

Multivariate analyses of associations between genetic subsets of the ELN-2017 categories, other risk factors and outcomes are shown in Supplemental Figures 12-14. Overall, our analyses support the revised risk group assignment of certain molecular subgroups as introduced in the ELN-2017 recommendations.

Potential further refinement of the ELN-2017 risk categories through inclusion of additional gene mutations

When the gene mutations listed in Table 1 were individually included in the multivariate analyses shown in Figure 4, only *DNMT3A* mutations significantly associated with inferior RFS as well as OS, with an approximately 1.3-fold risk increase for either outcome (Supplemental

Figure 19). Within each ELN-2017 risk category, mutated *DNMT3A* identified a subgroup with significantly inferior OS compared to *DNMT3A* wild-type patients (Supplemental Figure 20).

Supplemental Table 1: Reasons for patient re-classification into a higher or lower risk category according to ELN-2017 as compared to ELN-2010.

ELN-2010 genetic group	ELN-2017 risk group	Reason for reclassification	N
**Favorable**	**Intermediate**	Cytogenetically normal AML, monoallelic *CEBPA* mutation	12
**Favorable**	**Adverse**	Cytogenetically normal AML, monoallelic *CEBPA* mutation and mutated *RUNX1* and/or *ASXL1*[#]	7[#]
**Intermediate-I**	**Favorable**	Cytogenetically normal AML, mutated *NPM1* and *FLT3*-ITD with low allelic ratio	24
**Intermediate-I**	**Adverse**	Cytogenetically normal AML, *FLT3*-ITD with high allelic ratio	16[†]
		Cytogenetically normal AML, mutated *RUNX1* and/or *ASXL1*	64
		Cytogenetically normal AML, mutated *TP53*	3
**Intermediate-II**	**Favorable**	Non-normal cytogenetics, biallelic CEBPA mutation	4
		Non-normal cytogenetics, mutated *NPM1* and *FLT3*-ITD negative or low allelic ratio	19
**Intermediate-II**	**Adverse**	Non-normal cytogenetics, *FLT3*-ITD with high allelic ratio	11[‡]
		Non-normal cytogenetics, mutated *RUNX1* and/or *ASXL1*	42
		Non-normal cytogenetics, mutated *TP53*	2

Footnotes:

# Two of these patients also had a FLT3-ITD with high allelic ratio, and one also had mutated *TP53*.

† Six of these patients also had mutated ASXL1 and/or RUNX1, and one also had mutated *TP53*

‡ Four of these patients also had mutated RUNX1

Supplemental Table 2: Baseline patient characteristics

**Variable**<br>Patient number	**Incidence cohort**<br>n=771	**Outcomes cohort**<br>n=1116
**Age** [years], median (range)	57 (18-86)	58 (18 – 86)
**Male sex**	388 (50%)	571 (51%)
**Disease type**		
De novo AML	654 (85%)	936 (84%)
Secondary AML	73 (9%)	123 (11%)
Therapy-related AML	44 (6%)	57 (5%)
**WBC** [×10^{9}/L], median (range)	19.8 (0.46 – 486)	20.2 (0.46 – 486)
**Bone marrow blasts [%],** median (range)	80 (6 – 100)	80 (6 – 100)
**MRC cytogenetic risk category**		
Favorable	81 (11%)	81 (7%)
Intermediate	530 (69%)	875 (78%)
Adverse	160 (21%)	160 (14%)
**ELN 2010 genetic group**[#]		
Favorable,	244 (32%)	367 (33%)
Intermediate-I	221 (29%)	443 (40%)
Intermediate-II	142 (18%)	142 (13%)
Adverse	164 (21%)	164 (15%)
**ELN 2017 prognostic group**		
Favorable,	272 (35%)	422 (38%)
Intermediate	190 (25%)	295 (26%)
Adverse	309 (40%)	399 (36%)
**Gene mutations detected in pretreatment sample**		
NPM1	244 (32%)	431 (39%)
FLT3-ITD	195 (25%)	303 (27%)
- low allelic ratio	77	131
- high allelic ratio	118	172
CEBPA	66 (9%)	82 (7%)
- mono-allelic	35 (5%)	45 (4%)
- bi-allelic	31 (4%)	37 (3%)
RUNX1	114 (15%)	178 (16%)
ASXL1	90 (12%)	135 (12%)
TP53	75 (10%)	80 (7%)

Abbreviations: WBC, white blood cell count; FAB, French-American British classification; MRC, British Medical Research Council; ELN, European LeukemiaNet; ITD, internal tandem duplication

Supplemental Table 3: Characteristics of patients aged <60 years achieving CR according to postremission treatment received in first remission

	**Postremission treatment**			
**Variable**<br>Patient number	**Cytotoxic therapy only**<br>n=240	**Autologous SCT in CR1**<br>n=44	**Allogeneic SCT in CR1**<br>n=97	***P***
**Age** [years], median (range)	45 (19 – 59)	45 (18 – 59)	42 (18 – 59)	.044
**Male sex**	111 (46%)	25 (57%)	39 (40%)	.19
**Disease type**				.08
De novo AML	226 (94%)	43 (98%)	85 (88%)	
Secondary AML	10 (9%)	1 (2%)	6 (6%)	
Therapy-related AML	4 (2%)	0	6 (6%)	
**WBC** [$\times 10^9$/L], median (range)	23.1 (0.8 – 486)	21.6 (1.0 – 391)	20.5 (0.8 – 231)	.79
**Bone marrow blasts [%],** median (range)	80 (13 – 100)	85 (20 – 100)	80 (19 – 100)	.05
**ELN 2017 risk group**				.06
Favorable	132 (55%)	24 (55%)	40 (41%)	
Intermediate	72 (30%)	10 (23%)	31 (32%)	
Adverse	36 (15%)	10 (23%)	26 (27%)	
**MRC cytogenetic risk category**				.15
Favorable	32 (13%)	8 (18%)	6 (6%)	
Intermediate	189 (79%)	34 (77%)	80 (82%)	
Adverse	19 (8%)	2 (5%)	11(11%)	

Abbreviations: WBC, white blood cell count; ELN, European LeukemiaNet; MRC, British Medical Research Council.

Supplemental Table 4: Outcomes according to genetic subsets within the ELN-2017 risk groups

ELN 2017 genetic risk group	Complete remission		RFS		OS	
	**n [%]**	***P***	**5-year RFS, % (95% CI)**	***P***	**5-year OS, % (95% CI)**	***P***
**Favorable (n=422)**						
inv(16)/t(16;16) (n=45)	37 (82)	.19	63.7 (49.7-81.6)	.25	70.3 (58.0-85.3)	.010
t(8;21) (n=36)	21 (58)		61.9 (44.3-86.6)		50.0 (36.1-69.3)	
Biallelic mutated CEBPA (n=37)	26 (70)		60.3 (43.8-83.0)		69.7 (56.2-86.4)	
NPM1^{mut}, *FLT3*-ITD^{neg} (n=238)	175 (74)		49.7 (42.6-57.9)		50.9 (44.8-57.8)	
NPM1^{mut}, *FLT3*-ITD^{low} (n=66)	46 (70)		51.9 (39.2-68.7)		47.6 (36.9-61.5)	
**Intermediate (n=295)**						
NPM1^{mut}, *FLT3*-ITD^{high} (n=119)	79 (66)	.90	27.9 (19.5-40.0)	.024	28.5 (21.3-38.0)	.10
NPM1^{wt}, *FLT3*-ITD^{low} (n=26)	17 (65)		5.9 (0.9-39.4)		36.4 (21.7-61.2)	
t(9;11) (n=22)	13 (59)		36.9 (17.8-76.8)		20.5 (8.7-48.1)	
Other intermediate features (n=128)	86 (67)		26.4 (18.4-38.0)		33.2 (25.8-42.8)	
**Adverse (n=399)**						
t(v;11q23.3) (n=24)	11 (46)	.19	37.5 (16.2-86.8)	<.0001	44.3 (28.0-70.0)	<.0001
ASXL1^{mut} and/or *RUNX*^{mut} (n=184)	82 (45)		10.8 (5.8-20.3)		11.6 (7.7-17.5)	
NPM1^{wt}, *FLT3*-ITD^{high} (n=23)	11 (48)		9.1 (1.4-58.9)		8.7 (2.3-32.7)	
Complex karyotype and *TP53*^{wt} (n=30)	15 (50)		20.0 (7.4-55.0)		22.9 (11.7-44.5)	
Complex karyotype and *TP53*^{mut} (n=62)	17 (27)		0 (not defined)		0 (not defined)	
Other adverse features (n=33)	14 (42)		15.5 (4.3-55.3)		11.8 (4.4-31.5)	
Multiple adverse features (n=43)	14 (33)		7.9 (1.2-51.5)		9.9 (3.9-25.0)	

Abbreviations: ELN, European LeukemiaNet; RFS, relapse-free survival; OS, overall survival; CI, confidence interval; ITD, internal tandem duplication.

Supplemental Table 5: Outcomes according to the proposed refined ELN-2017 genetic risk categories

Refined ELN 2017 genetic risk group	Complete remission		RFS		OS	
	n [%]	*P*	5-year RFS, % (95% CI)	*P*	5-year OS, % (95% CI)	*P*
**All patients (n=1116)**						
Very favorable (n=82)	63 (77)	<.0001	62.1 (50.9-75.7)	<.0001	70.1 (60.7-80.9)	<.0001
Favorable (n=340)	242 (71)		51.2 (45.1-58.0)		50.2 (45.1-55.9)	
Intermediate (n=295)	195 (66)		25.8 (20.2-32.9)		30.6 (25.7-36.5)	
Adverse (n=337)	147 (44)		13.4 (8.7-20.5)		14.3 (10.9-18.7)	
Very adverse (n=62)	17 (27)		0 (not defined)		0 (not defined)	
**Age <60 years (n=599)**						
Very favorable (n=63)	52 (83)	<.0001	65.7 (53.6-80.5)	<.0001	77.2 (67.4-88.5)	<.0001
Favorable (n=198)	144 (73)		61.4 (53.8-70.1)		60.1 (53.6-67.4)	
Intermediate (n=171)	113 (66)		36.6 (28.6-46.8)		41.5 (34.6-49.7)	
Adverse (n=151)	67 (44)		24.1 (15.7-37.0)		21.9 (16.1-29.8)	
Very adverse (n=16)	5 (31)		0 (not defined)		0 (not defined)	
**Age ≥60 years (n=517)**						
Very favorable (n=19)	11 (58)	<.0001	45.5 (23.8-86.8)	<.0001	47.4 (29.5-76.1)	<.0001
Favorable (n=142)	98 (69)		36.0 (27.5-47.2)		35.1 (28.1-45.1)	
Intermediate (n=124)	82 (66)		11.3 (6.1-21.0)		16.0 (10.6-24.2)	
Adverse (n=186)	80 (43)		4.3 (1.3-13.8)		8.0 (4.8-13.3)	
Very adverse (n=46)	12 (26)		0 (not defined)		0 (not defined)	

Abbreviations: ELN, European LeukemiaNet; RFS, relapse-free survival; OS, overall survival; CI, confidence interval; ITD, internal tandem duplication.

Supplemental Figure 1: Patient disposition in the AMLCG-1999 and AMLCG-2008 trials

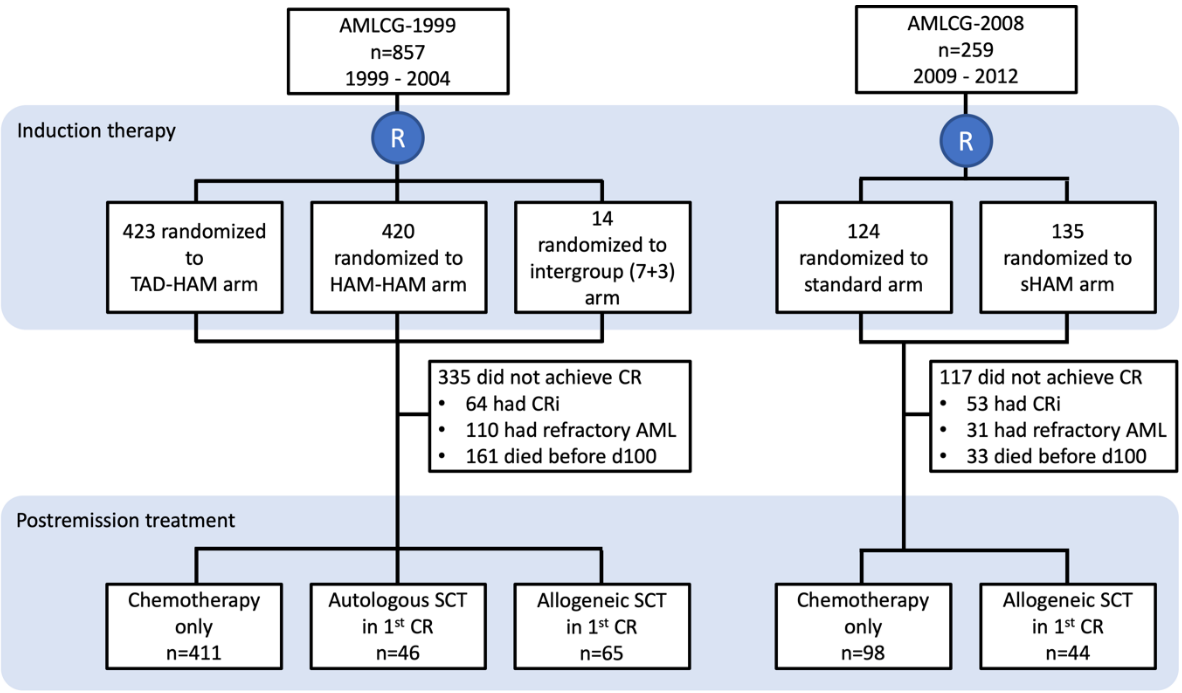

Supplemental Figure 2: Association between the ELN-2017 risk groups and clinical parameters

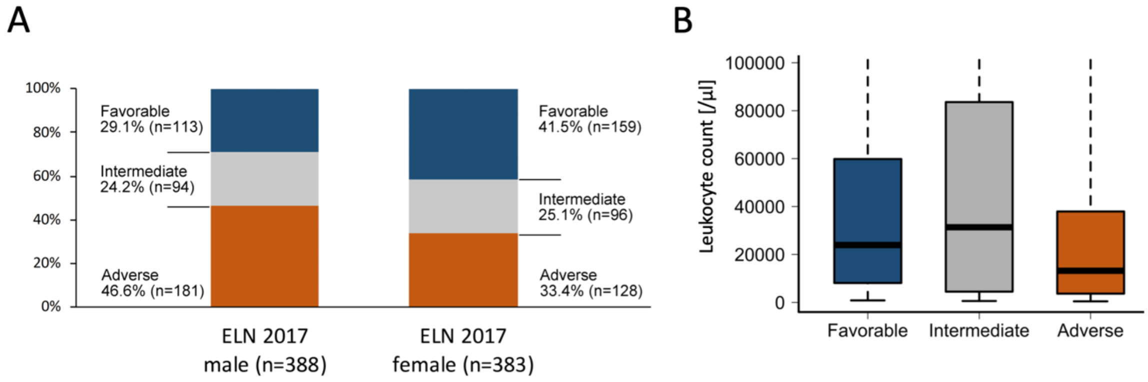

(A) Distribution of the ELN-2017 risk categories in male and female AML patients. **(B)** Peripheral blood leukocyte counts according to ELN-2017 genetic risk group.

Supplemental Figure 3: Time-dependent areas under the receiver operating characteristic curves (AUC) for the association between the ELN-2017 risk groups and survival outcomes

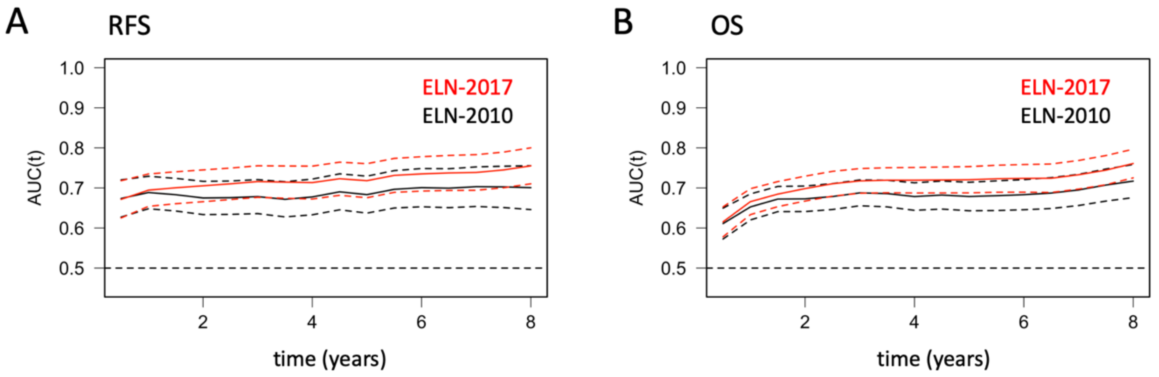

The plots show time-dependent AUCs for **(A)** RFS and **(B)** OS, calculated at 6-month intervals from the time of AML diagnosis. The red solid and dashed lines represent the time-dependent AUC for the ELN-2017 risk classification and its confidence interval, respectively, and the black lines show the AUC and its confidence interval for the ELN-2010 groups. At each time point, the marker with the higher AUC performs better in correctly predicting the survival outcomes of individual patients at that time. Receiver operating characteristic curves were calculated using R package "timeROC".[8]

With the exception of the first analyzed time point (6 months after AML diagnosis), the ELN-2017 classification provided a better risk prediction for RFS and OS than the ELN-2010 classification. For RFS, the difference between the AUCs was statistically significant (P<.05) at most time points beyond 2.5 years. For OS, the difference in the AUCs was statistically significant at two years from initial diagnosis and at all later time points.

Supplemental Figure 4: Outcomes of patients who were re-assigned into a higher or lower risk category by the ELN-2017 guidelines compared to the ELN-2010 genetic groups

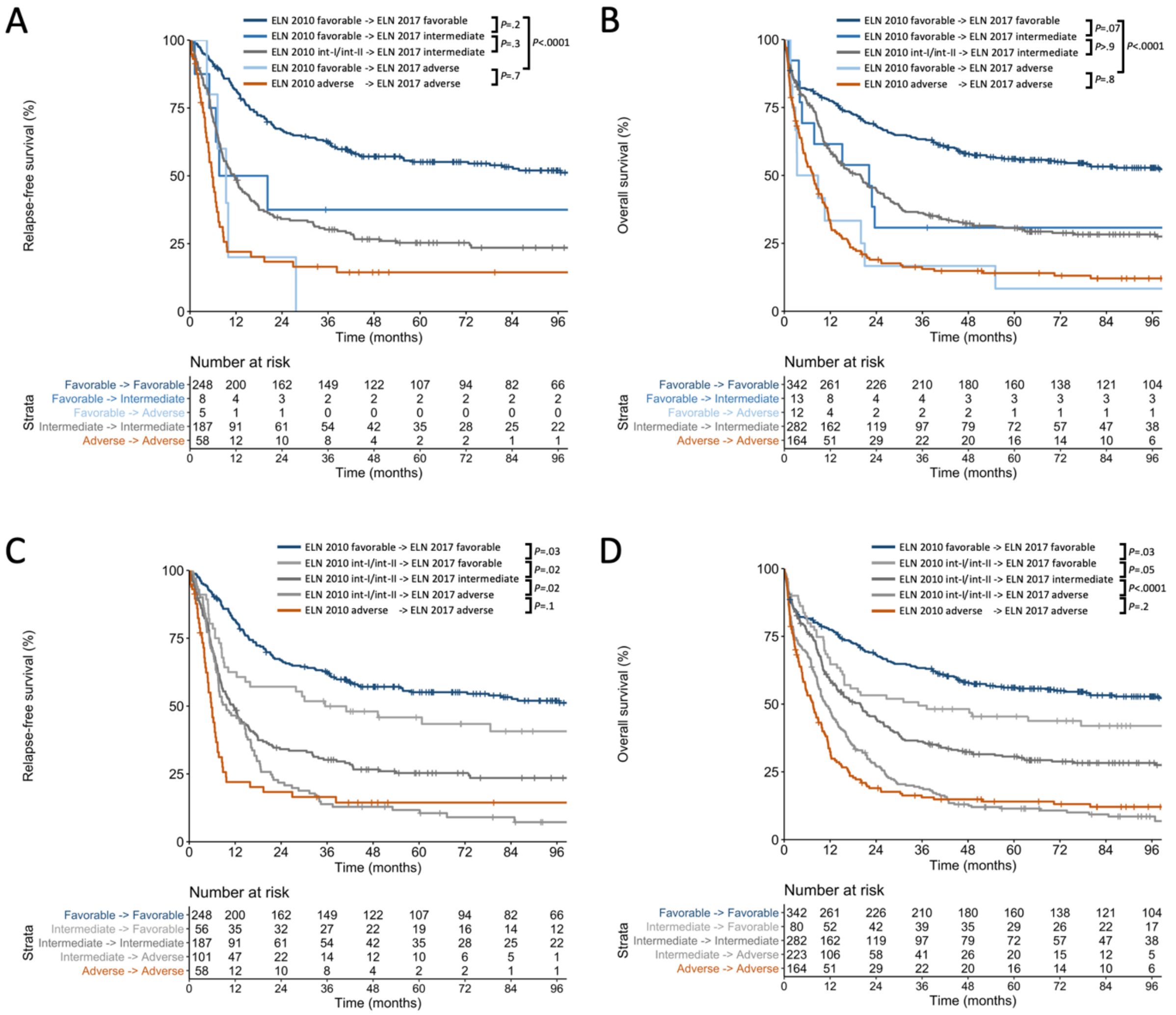

(A) Relapse-free survival and **(B)** overall survival of ELN-2010 favorable-risk patients who were classified as favorable-, intermediate-, or adverse-risk according to the ELN-2017 guidelines, in comparison to the patients classified as intermediate- or adverse-risk by both classification systems. Of 367 patients classified as favorable according to ELN-2010, 342 were classified as favorable, 13 as intermediate and 12 as adverse-risk by the ELN-2017 classification. The 13 patients re-classified into the intermediate group showed no significant difference in RFS (*P*=.2) but had a trend toward shorter OS (*P*=.07) compared to patients who remained in the favorable category. Their outcomes were similar to patients who were intermediate-risk in both classifications. The 12 patients re-classified into the adverse group had a significantly shorter RFS and OS compared to patients who remained in the favorable category (*P*<.0001 for both endpoints) and had outcomes similar to patients who were adverse-risk in both classifications.

(C) Relapse-free survival and **(D)** overall survival of ELN-2010 intermediate-I or intermediate-II-risk patients who were classified as favorable-, intermediate-, or adverse-risk according to the ELN-2017 guidelines, in comparison to the patients classified as favorable- or adverse-risk by both classification systems. Of the 585 ELN-2010 intermediate-I/II patients, 80 were re-classified as favorable, 282 remained intermediate and 223 were assigned to the adverse-risk category by the ELN-2017 classification. The 80 patients re-classified into the favorable group had significantly longer RFS (P=.02) and OS (P=.05) compared to the 213 patients who were re-classified as intermediate. Of note, these 80 patients still had shorter RFS (P=.03) and OS (P=.03) compared patients who were favorable in both the ELN-2010 and ELN-2017 system. The 223 patients re-classified into the adverse group had significantly shorter RFS (P=.02) and OS (P<.0001) compared to patients who were classified as intermediate in both the ELN-2010 and 2017 guidelines. Their RFS and OS were similar to patients who were classified as adverse-risk by both classifications.

Supplemental Figure 5: Postremission therapy of patients who achieved CR after protocol-specified induction therapy

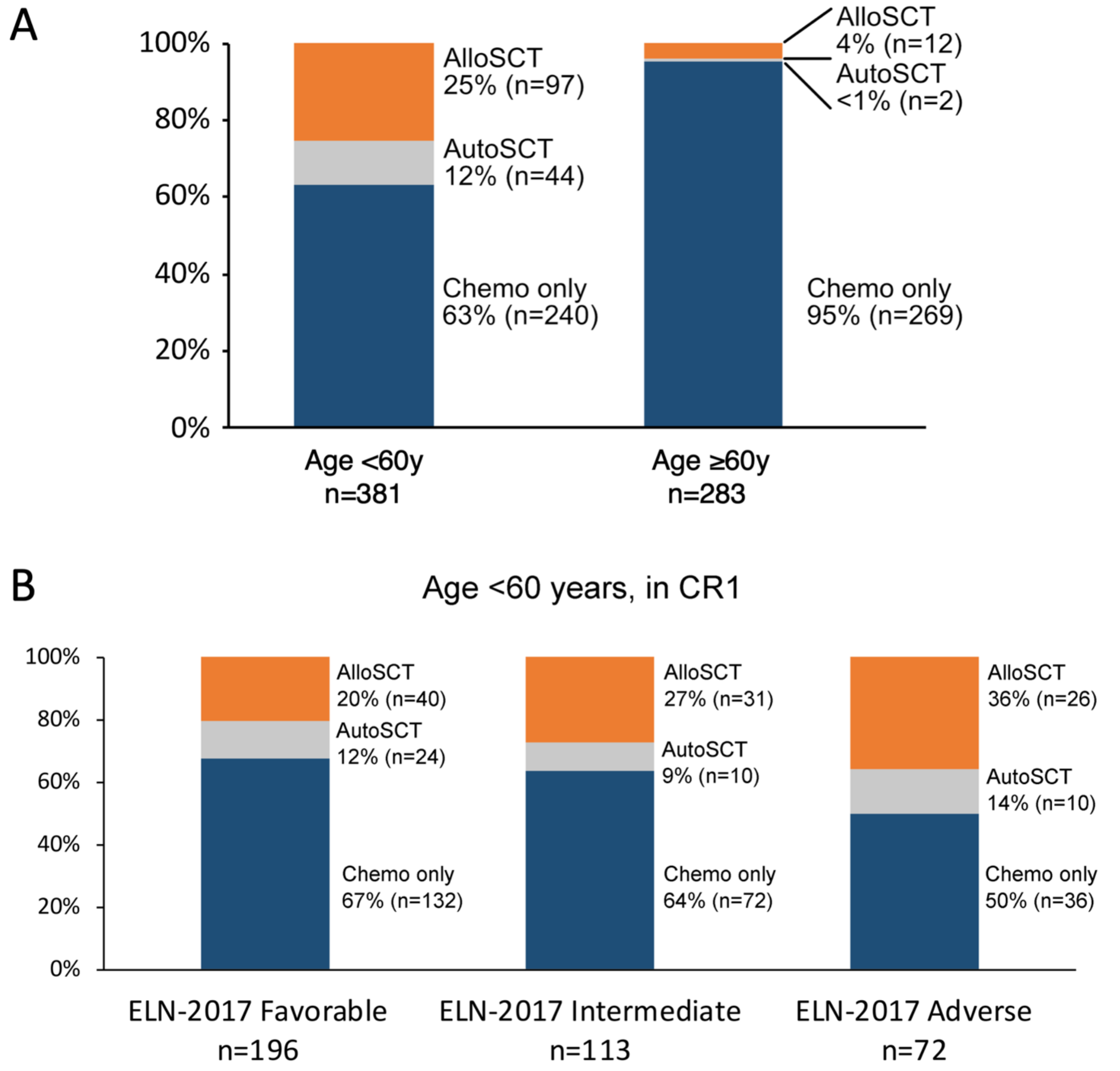

(A) Postremission therapy received in CR1 according to patient age. **(A)** Postremission therapy in CR1 in patients aged <60 years according to ELN-2017 risk category.

Supplemental Figure 6: Outcomes of ELN-2017 favorable-risk patients aged <60 years according to postremission treatment received in CR1

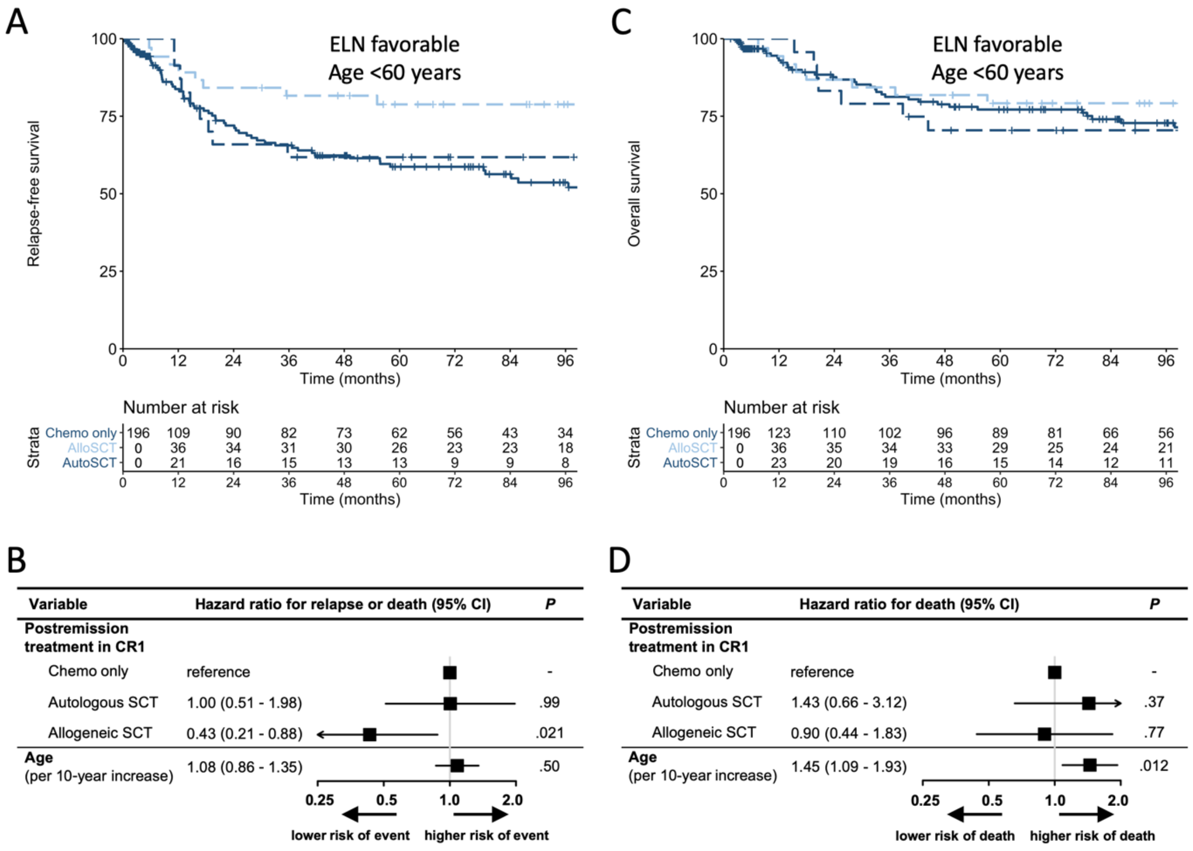

(A) Simon-Makuch plot of relapse-free survival according to postremission treatment received in CR1. **(B)** Forrest plot showing relapse-free survival according to postremission treatment, adjusted for age. **(C)** Simon-Makuch plot of overall survival according to postremission treatment received in CR1. **(D)** Forrest plot showing overall survival according to postremission treatment, adjusted for age. In the multivariable models in Panels B and D, transplantation was treated as time-dependent covariable.

Supplemental Figure 7: Outcomes of ELN-2017 intermediate-risk patients aged <60 years according to postremission treatment received in CR1

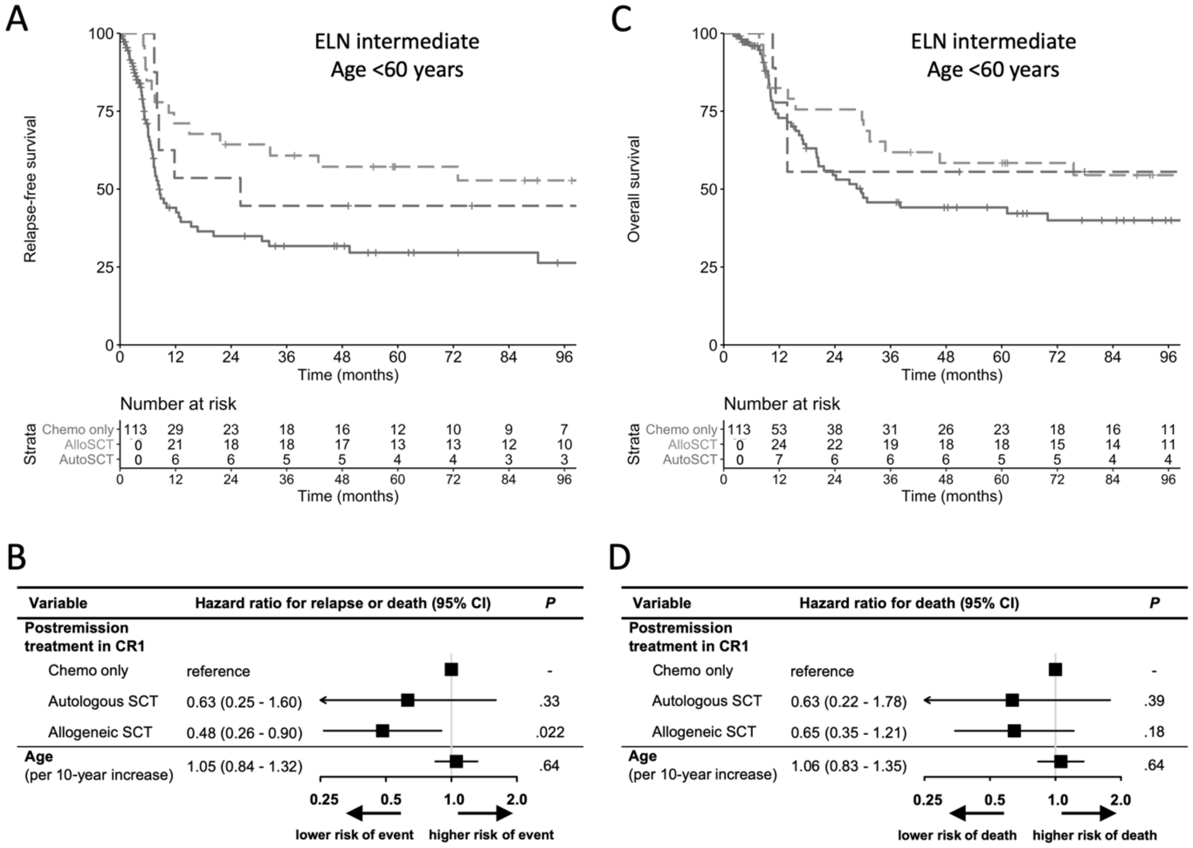

(A) Simon-Makuch plot of relapse-free survival according to postremission treatment received in CR1. **(B)** Forrest plot showing relapse-free survival according to postremission treatment, adjusted for age. **(C)** Simon-Makuch plot of overall survival according to postremission treatment received in CR1. **(D)** Forrest plot showing overall survival according to postremission treatment, adjusted for age. In the multivariable models in Panels B and D, transplantation was treated as time-dependent covariable.

Supplemental Figure 8: Outcomes of ELN-2017 adverse-risk patients aged <60 years according to postremission treatment received in CR1

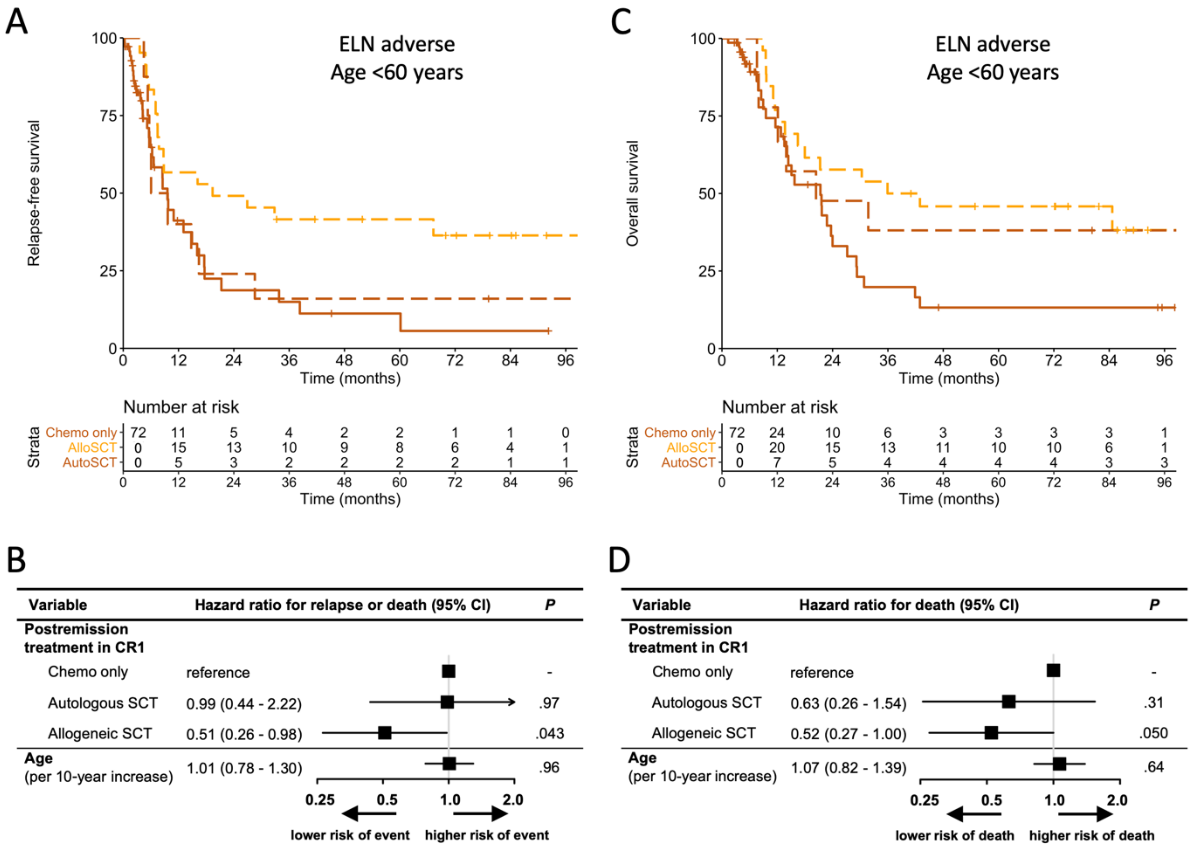

(A) Simon-Makuch plot of relapse-free survival according to postremission treatment received in CR1. **(B)** Forrest plot showing relapse-free survival according to postremission treatment, adjusted for age. **(C)** Simon-Makuch plot of overall survival according to postremission treatment received in CR1. **(D)** Forrest plot showing overall survival according to postremission treatment, adjusted for age. In the multivariable models in Panels B and D, transplantation was treated as time-dependent covariable.

Supplemental Figure 9: Outcomes of specific genetic subsets with the ELN-2017 risk categories

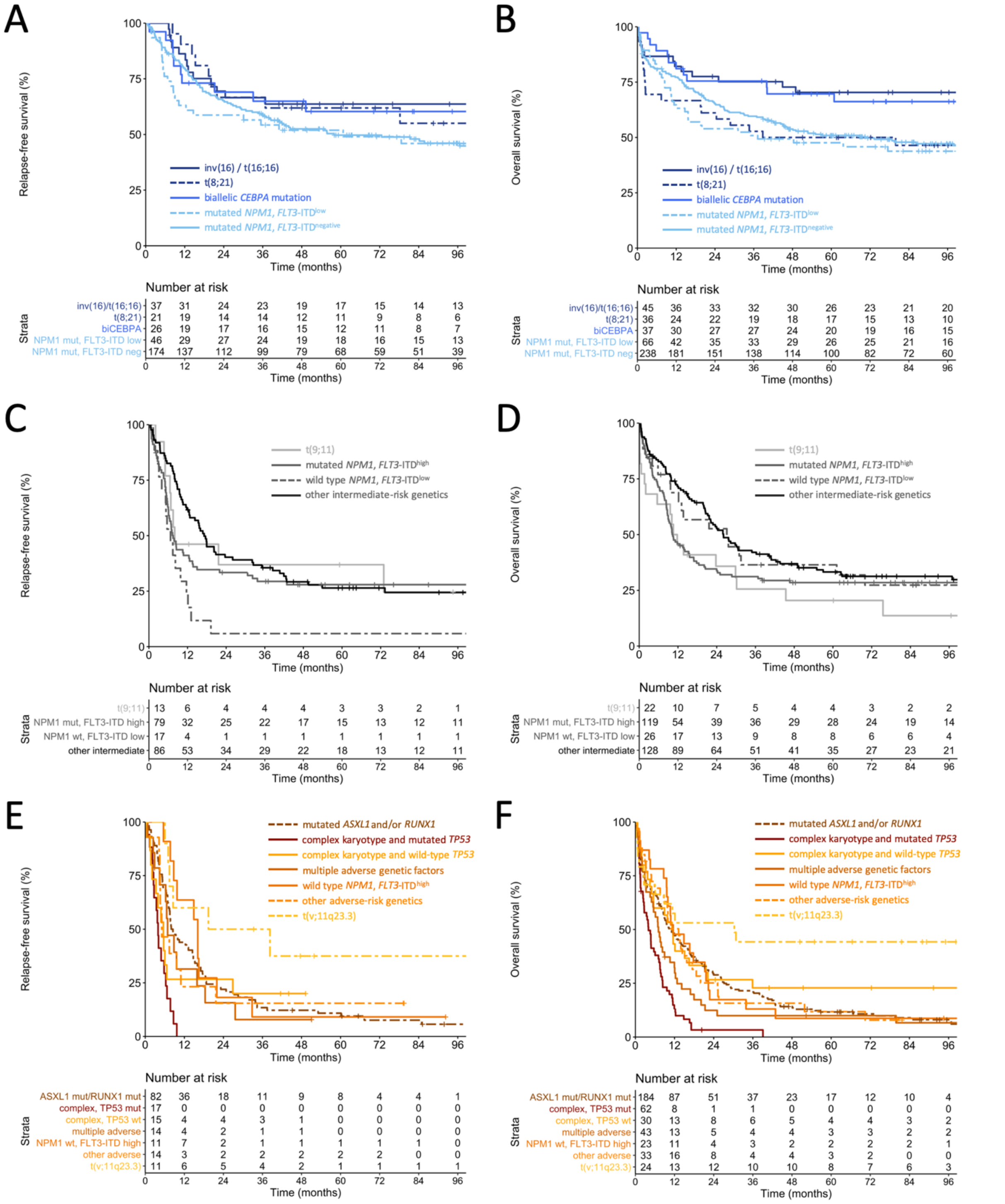

(A) Relapse-free survival and **(B)** overall survival of genetic subgroups within the ELN-2017 favorable-risk category. **(C)** Relapse-free survival and **(D)** overall survival of genetic subgroups within the ELN-2017 intermediate-risk category. Patients with wild-type *NPM1* and no *FLT3*-ITD are included in the “other intermediate” category. **(E)** Relapse-free survival and **(F)** overall survival of genetic subgroups within the ELN-2017 adverse-risk category.

Supplemental Figure 10: Outcomes of ELN-2017 adverse-risk patients according to *ASXL1* and *RUNX1* mutation status

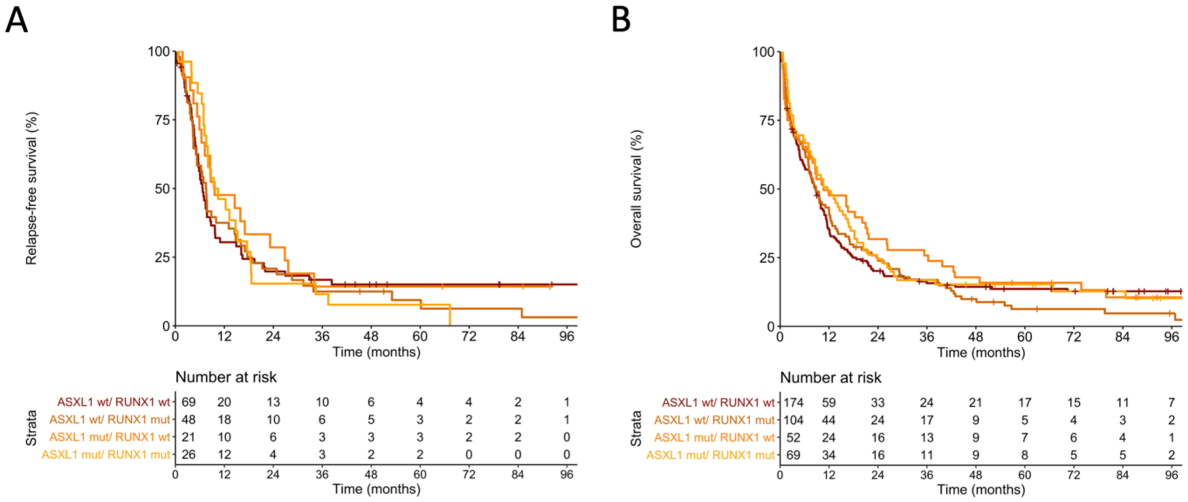

(A) Relapse-free survival and **(B)** overall survival in 399 ELN-2017 adverse-risk patients.

Supplemental Figure 11: Outcomes of patients according to *NPM1* mutations status and *FLT3*-ITD status and mutant-to-wild type allelic ratio

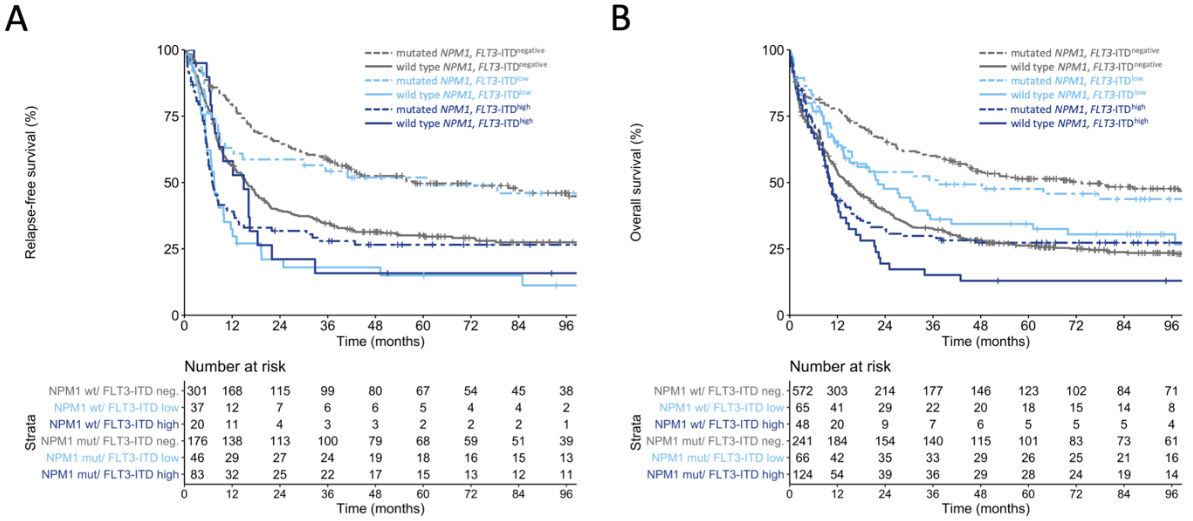

(A) Relapse-free survival and **(B)** overall survival in the entire cohort of 1116 patients (age range, 18-86 years), not taking other molecular markers into account. '*FLT3*-ITD low' designates a mutant-to-wild type ratio of<0.5; *FLT3*-ITD high designates a mutant-to-wild type ratio ≥0.5.

Supplemental Figure 12: Multivariate analysis of factors associated with achievement of complete remission, considering specific genetic subsets with the ELN-2017 risk categories and other pretreatment prognostic variables

Variable	Odds ratio for complete remission (95% CI)	*P*
**ELN-2017 genetic subset**		
Favorable		
inv(16)/t(16;16)	2.36 (0.96 - 5.81)	.061
t(8;21)	0.60 (0.27 - 1.33)	.21
biallelic CEBPA mut.	1.15 (0.51 - 2.63)	.73
NPM1^{mut}, *FLT3*-ITD^{neg}	1.45 (0.89 - 2.37)	.14
NPM1^{mut}, *FLT3*-ITD^{low}	1.18 (0.60 - 2.31)	.63
Intermediate		
NPM1^{mut}, *FLT3*-ITD^{high}	1.29 (0.72 - 2.32)	.39
NPM1^{wt}, *FLT3*-ITD^{low}	0.82 (0.32 - 2.05)	.67
t(9;11)	0.90 (0.33 - 2.46)	.84
Other intermediate features	reference	-
Adverse		
t(v;11q23.3)	0.45 (0.18 - 1.16)	.10
ASXL1^{mut} and/or *RUNX*^{mut}	0.46 (0.28 - 0.75)	.0018
NPM1^{wt}, *FLT3*-ITD^{high}	0.71 (0.27 - 1.90)	.50
Complex karyotype, *TP53*^{wt}	0.48 (0.20 - 1.13)	.093
Complex karyotype, *TP53*^{mut}	0.20 (0.10 - 0.40)	<.0001
Other adverse features	0.34 (0.15 - 0.77)	.010
Multiple adverse features	0.22 (0.10 - 0.48)	.0001
**Age** (per 10-year increase)	0.86 (0.77 - 0.96)	.0052
**Female sex**	1.08 (0.82 - 1.42)	.58
**WBC** (per 50,000/µl increase)	0.75 (0.66 - 0.84)	<.0001
***de novo* AML**	reference	-
**secondary AML**	0.47 (0.31 - 0.72)	.0005
**therapy–related AML**	0.39 (0.21 - 0.72)	.0027

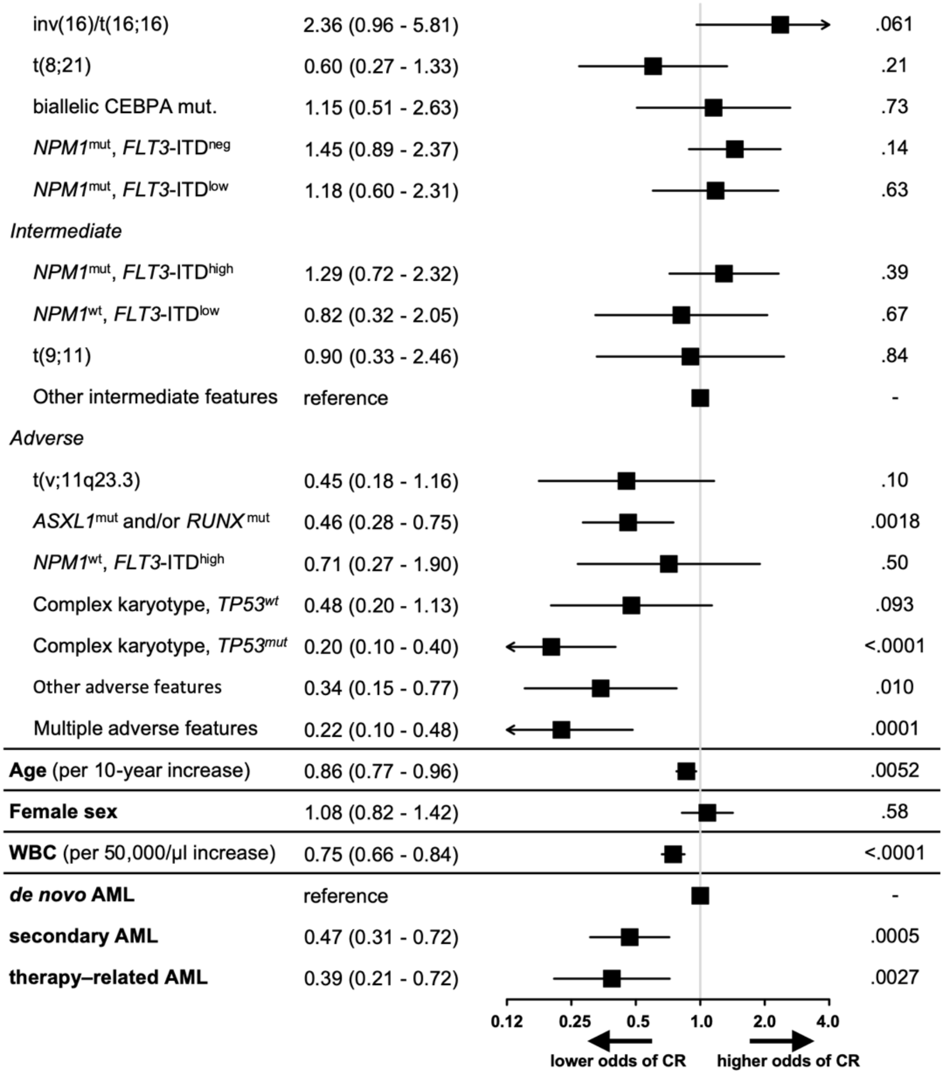

The multivariable model was stratified according to trial and induction therapy arm to account for potential differences in baseline risk between trials.

Supplemental Figure 13: Multivariate analysis of factors associated with relapse-free survival, considering specific genetic subsets with the ELN-2017 risk categories and other pretreatment prognostic variables

Variable	Hazard ratio for relapse or death (95% CI)	*P*
**ELN-2017 genetic subset**		
Favorable		
inv(16)/t(16;16)	0.40 (0.22 - 0.73)	.0027
t(8;21)	0.47 (0.23 - 0.96)	.038
biallelic CEBPA mut.	0.43 (0.22 - 0.84)	.013
NPM1^{mut}, *FLT3*-ITD^{neg}	0.52 (0.37 - 0.72)	.0001
NPM1^{mut}, *FLT3*-ITD^{low}	0.57 (0.36 - 0.92)	.021
Intermediate		
NPM1^{mut}, *FLT3*-ITD^{high}	1.07 (0.73 - 1.58)	.73
NPM1^{wt}, *FLT3*-ITD^{low}	3.12 (1.77 - 5.50)	.0001
t(9;11)	1.33 (0.64 - 2.72)	.44
Other intermediate features	reference	-
Adverse		
t(v;11q23.3)	1.13 (0.47 - 2.71)	.78
ASXL1^{mut} and/or *RUNX*^{mut}	1.62 (1.14 - 2.29)	.0067
NPM1^{wt}, *FLT3*-ITD^{high}	1.16 (0.58 - 2.34)	.67
Complex karyotype, *TP53*^{wt}	2.91 (1.47 - 5.73)	.0020
Complex karyotype, *TP53*^{mut}	4.85 (2.77 - 8.50)	<.0001
Other adverse features	1.93 (1.00 - 3.72)	.050
Multiple adverse features	1.91 (1.00 - 3.67)	.051
**Age** (per 10-year increase)	1.23 (1.13 - 1.34)	<.0001
**Female sex**	0.99 (0.81 - 1.21)	.92
**WBC** (per 50,000/µl increase)	1.17 (1.07 - 1.27)	.0009
***de novo* AML**	reference	-
**secondary AML**	1.11 (0.79 - 1.56)	.55
**therapy–related AML**	1.12 (0.67 - 1.87)	.67

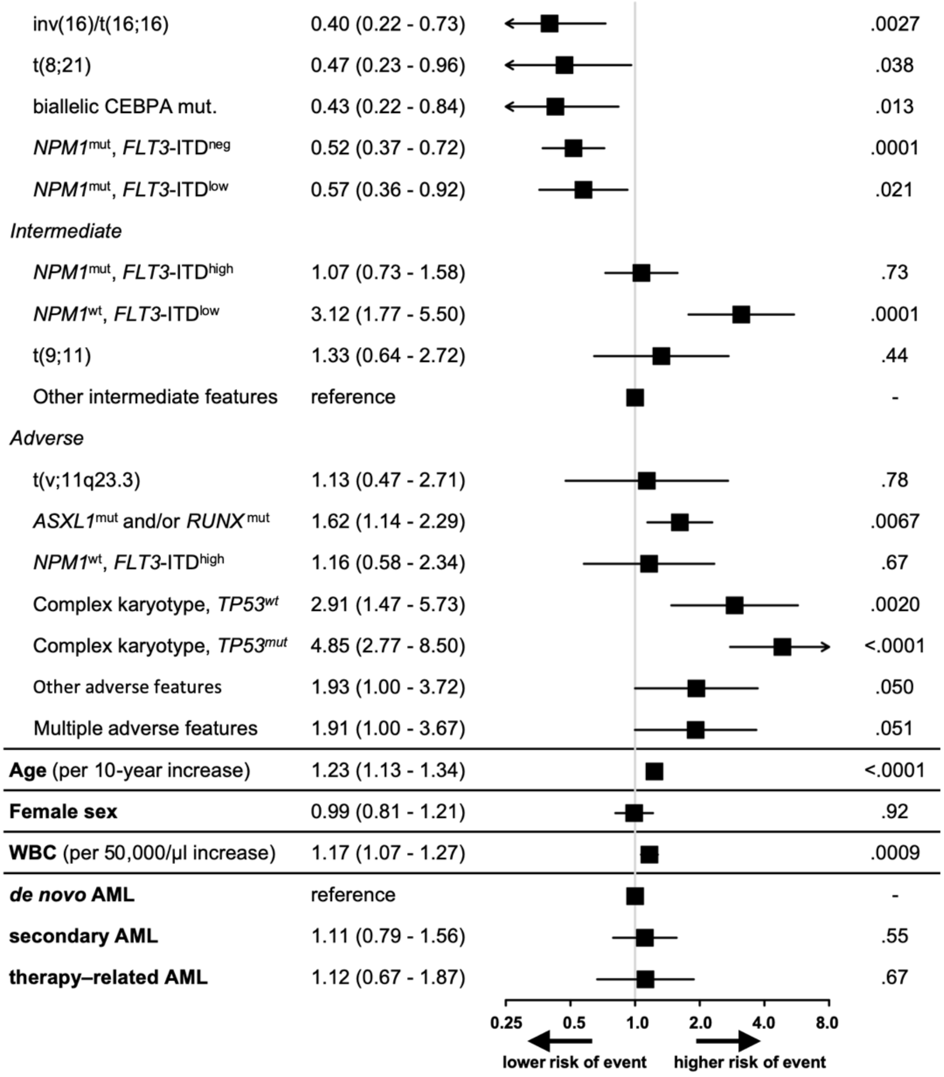

The multivariable model was stratified according to trial and induction therapy arm to account for potential differences in baseline risk between trials.

Supplemental Figure 14: Multivariate analysis of factors associated with overall survival, considering specific genetic subsets with the ELN-2017 risk categories and other pretreatment prognostic variables

Variable	Hazard ratio for death (95% CI)	*P*
**ELN-2017 genetic subset**		
Favorable		
inv(16)/t(16;16)	0.37 (0.21 - 0.67)	.0009
t(8;21)	0.87 (0.52 - 1.44)	.58
biallelic CEBPA mut.	0.47 (0.27 - 0.82)	.0008
NPM1^{mut}, *FLT3*-ITD^{neg}	0.66 (0.50 - 0.88)	.0041
NPM1^{mut}, *FLT3*-ITD^{low}	0.75 (0.51 - 1.10)	.15
Intermediate		
NPM1^{mut}, *FLT3*-ITD^{high}	1.17 (0.85 - 1.63)	.34
NPM1^{wt}, *FLT3*-ITD^{low}	1.34 (0.80 - 2.25)	.27
t(9;11)	2.11 (1.25 - 3.57)	.0052
Other intermediate features	reference	-
Adverse		
t(v;11q23.3)	1.49 (0.81 - 2.72)	.19
ASXL1^{mut} and/or *RUNX*^{mut}	1.63 (1.25 - 2.13)	.0003
NPM1^{wt}, *FLT3*-ITD^{high}	1.65 (1.01 - 2.69)	.045
Complex karyotype, *TP53*^{wt}	2.07 (1.27 - 3.37)	.0033
Complex karyotype, *TP53*^{mut}	3.63 (2.57 - 5.11)	<.0001
Other adverse features	1.93 (1.26 - 2.97)	.0027
Multiple adverse features	2.18 (1.49 - 3.19)	.0001
**Age** (per 10-year increase)	1.32 (1.24 - 1.41)	<.0001
**Female sex**	0.88 (0.75 - 1.02)	.09
**WBC** (per 50,000/µl increase)	1.18 (1.11 - 1.25)	<.0001
***de novo* AML**	reference	-
**secondary AML**	1.15 (0.91 - 1.44)	.24
**therapy–related AML**	1.61 (1.18 - 2.19)	.0026

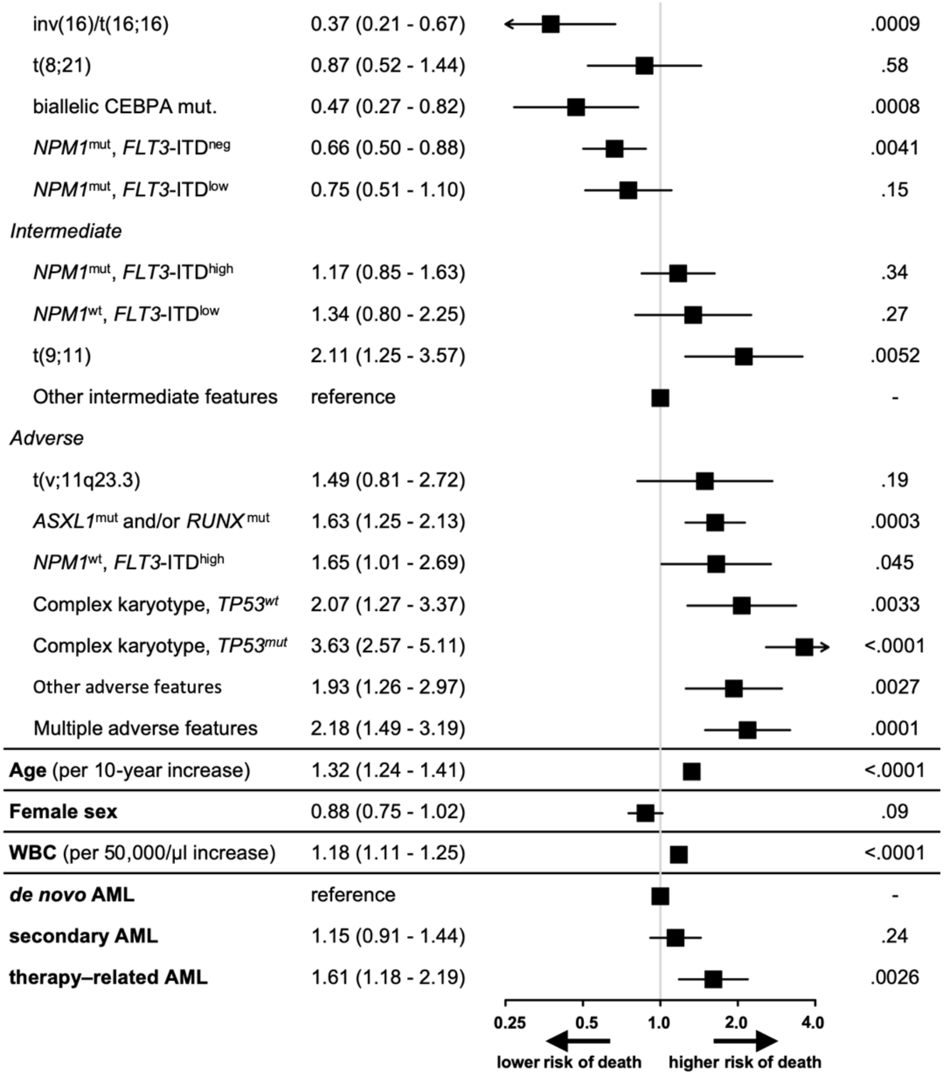

The multivariable model was stratified according to trial and induction therapy arm to account for potential differences in baseline risk between trials.

Supplemental Figure 15: Outcomes of patients according to the proposed refinement of the ELN-2017 genetic risk groups, stratified by age group

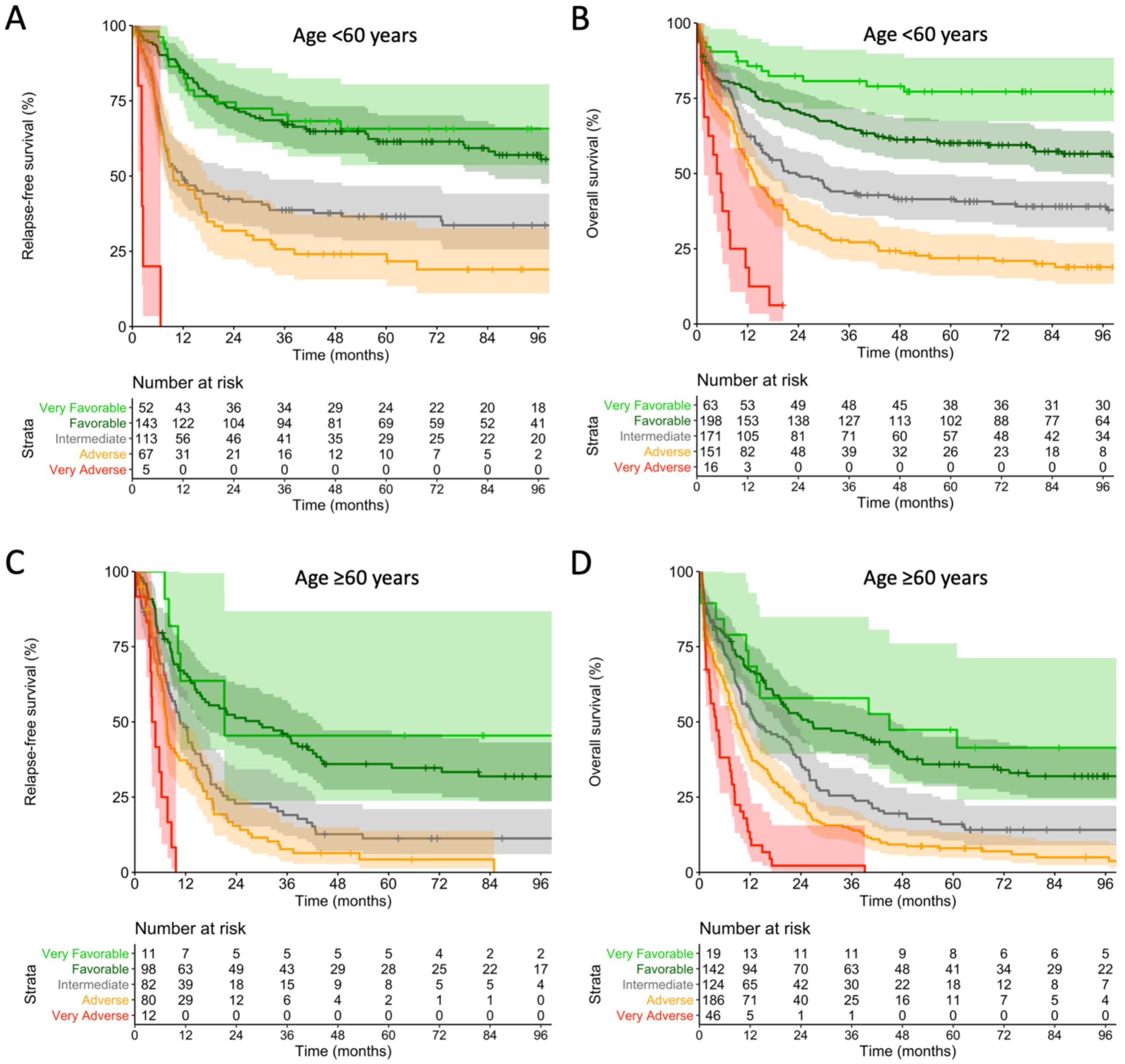

(A) Relapse-free survival and **(B)** overall survival according to the refined ELN-2017 categories in 599 patients aged <60 years. **(C)** Relapse-free survival and **(D)** overall survival according to the refined ELN-2017 categories in 517 patients aged ≥60 years.

Supplemental Figure 16: Multivariate analyses of outcomes according to the refined ELN-2017 genetic risk groups and other pretreatment variables

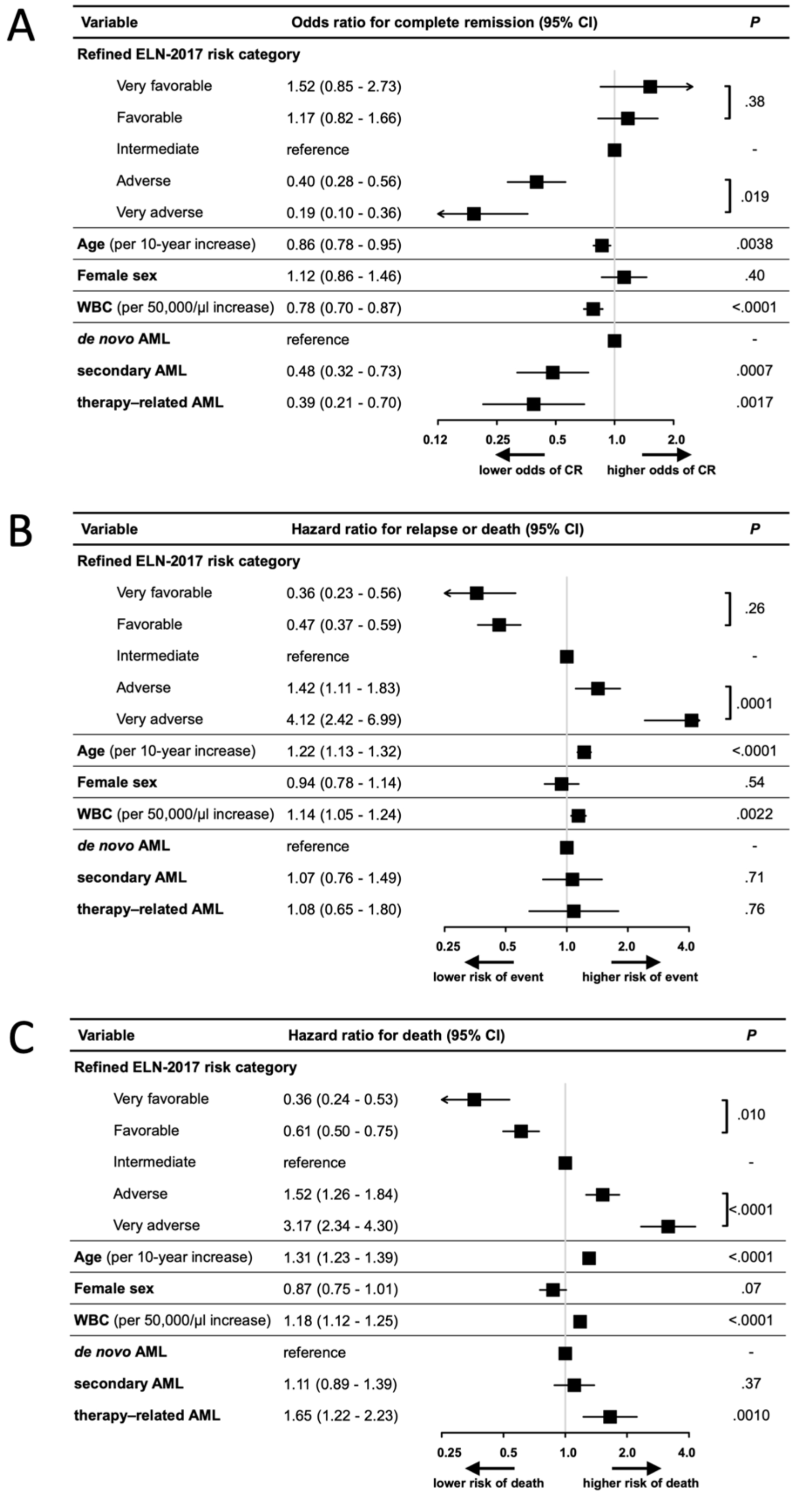

(A) Forrest plot showing odds ratios for achievement of complete remission. **(B)** Forrest plot showing hazard ratios for relapse-free survival. **(C)** Forrest plot showing hazard ratios for overall survival. The multivariable models were stratified according to trial and induction therapy arm to account for potential differences in baseline risk.

Supplemental Figure 17: Overall survival from the time of first relapse according to the refined ELN-2017 risk categories

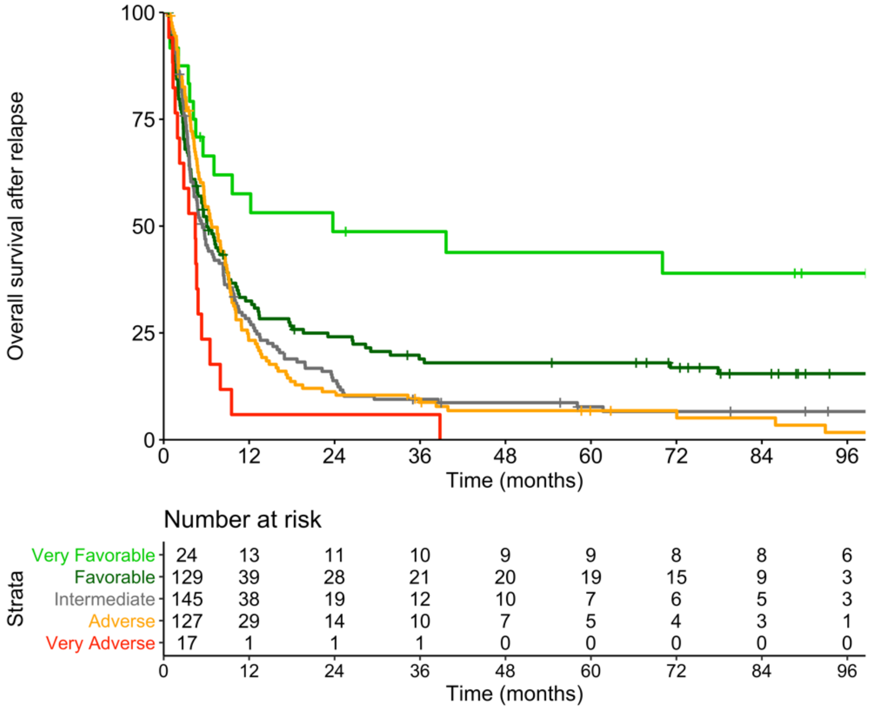

Overall survival 5 years after first relapse was 44% for the proposed “very favorable” category, 18% for the remaining “favorable” patients, 8% for the “intermediate” group, 7% for the “adverse” category and 0% for the proposed “very adverse” group.

Supplemental Figure 18: Validation of the proposed refinement of the ELN-2017 genetic risk groups in an independent, external cohort

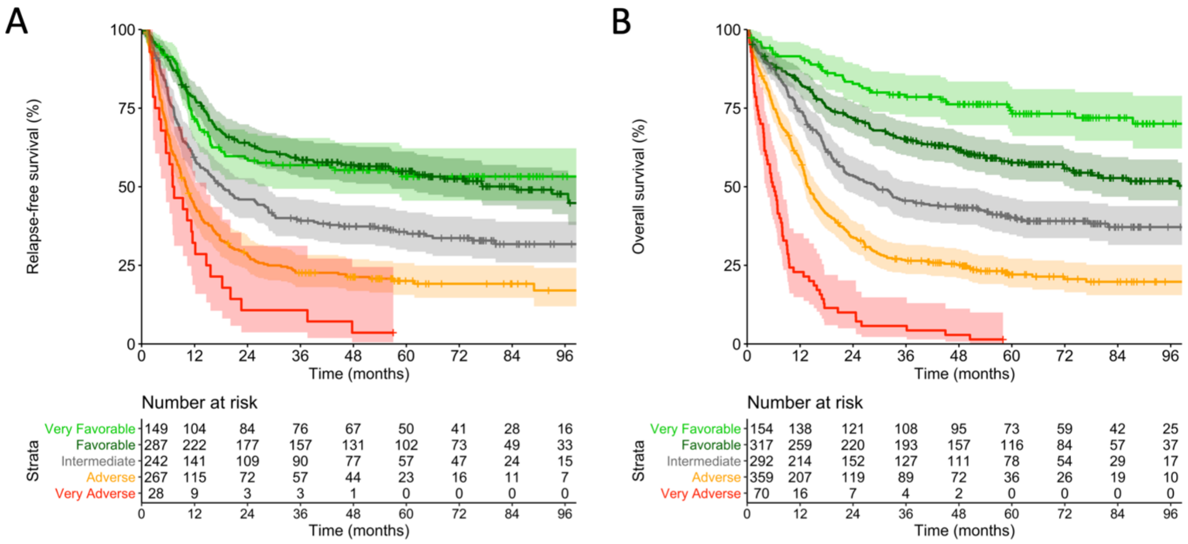

(A) Relapse-free survival and **(B)** overall survival according to the refined ELN-2017 categories in the AML-SG cohort. Overall survival at 5 years was 74% for the proposed “very favorable” category, 58% for the remaining “favorable” patients, 40% for the “intermediate” group, 22% for the “adverse” category and 1% for the proposed “very adverse” group. These data are very similar to the outcomes observed among younger patients (<60 years) in our own cohort (Supplemental Table 3).

Supplemental Figure 19: Multivariate analyses of outcomes according to the ELN-2017 genetic risk groups and other pretreatment prognostic variables, including *DNMT3A* mutation status

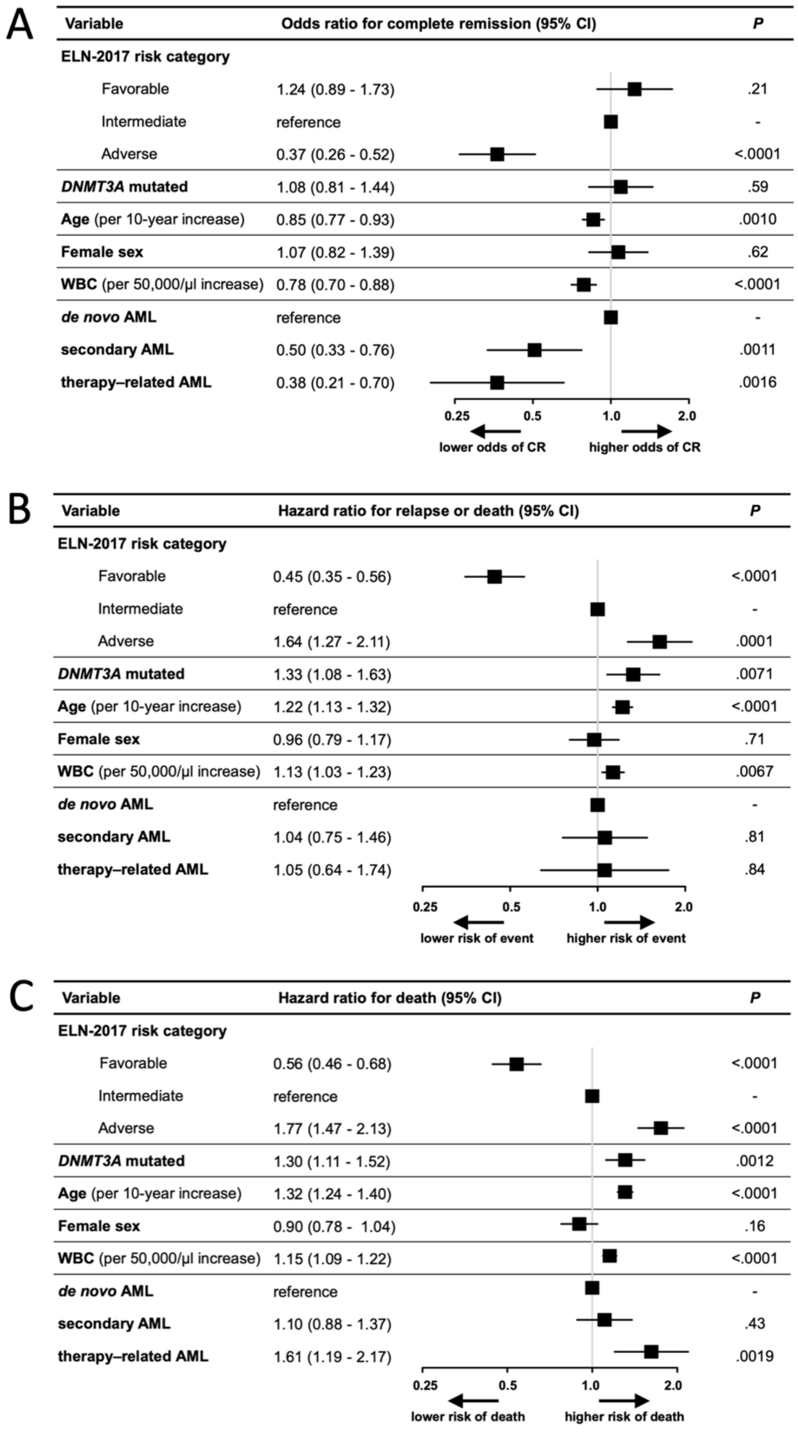

(A) Forrest plot showing odds ratios for achievement of complete remission. **(B)** Forrest plot showing hazard ratios for relapse-free survival. **(C)** Forrest plot showing hazard ratios for overall survival. The multivariable models were stratified according to trial and induction therapy arm to account for potential differences in baseline risk.

Supplemental Figure 20: Outcomes of patients according to the ELN-2017 genetic risk groups and *DNMT3A* mutation status

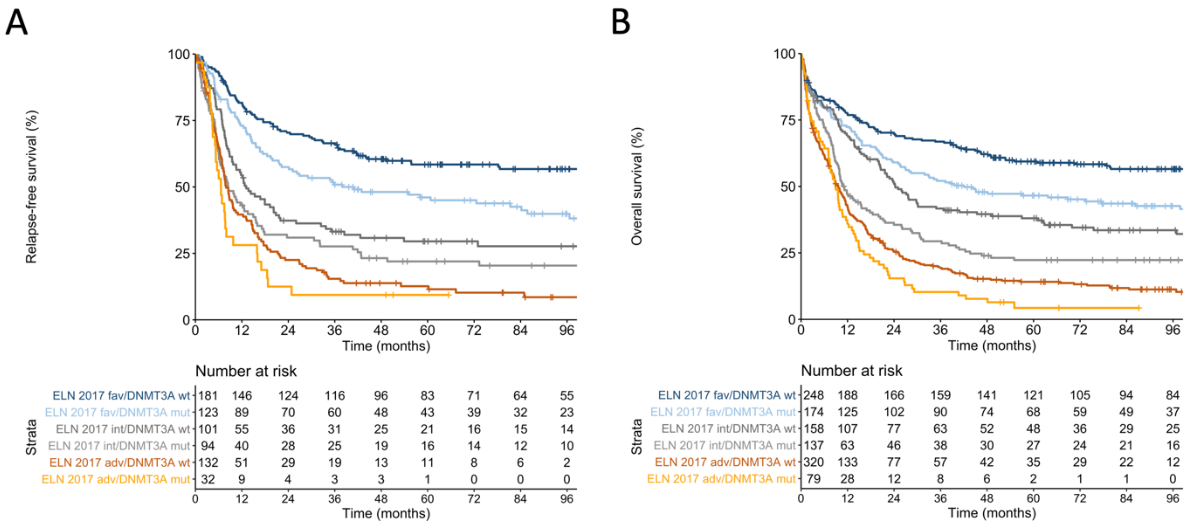

(A) Relapse-free survival and **(B)** overall survival according s in the entire cohort of 1116 patients (age range, 18-86 years).

Supplemental References

1. Büchner T, Berdel WE, Haferlach C, Haferlach T, Schnittger S, Müller-Tidow C et al. Age-related risk profile and chemotherapy dose response in acute myeloid leukemia: a study by the German Acute Myeloid Leukemia Cooperative Group. *J Clin Oncol* 2009; **27**: 61–69.
2. Büchner T, Schlenk RF, Schaich M, et al. Acute Myeloid Leukemia (AML): different treatment strategies versus a common standard arm- combined prospective analysis by the German AML Intergroup. *J Clin Oncol* 2012; **30**: 3604–3610.
3. Braess J, Amler S, Kreuzer K-A, Spiekermann K, Lindemann HW, Lengfelder E *et al.* Sequential high-dose cytarabine and mitoxantrone (S-HAM) versus standard double induction in acute myeloid leukemia-a phase 3 study. *Leukemia* 2018; **32**: 2558–2
4. Cheson BD, Bennett JM, Kopecky KJ, Büchner T, Willman CL, Estey EH *et al.* Revised recommendations of the International Working Group for Diagnosis, Standardization of Response Criteria, Treatment Outcomes, and Reporting Standards for Therapeutic Trials in Acute Myeloid Leukemia. *J Clin Oncol* 2003; **21**: 4642–4649.
5. Döhner H, Estey E, Grimwade D, Amadori S, Appelbaum FR, Büchner T et al. Diagnosis and management of AML in adults: 2017 ELN recommendations from an international expert panel. *Blood* 2017; **129:** 424–447.
6. Angenendt L, Röllig C, Montesinos P, et al. Chromosomal Abnormalities and Prognosis in NPM1-Mutated Acute Myeloid Leukemia: A Pooled Analysis of Individual Patient Data From Nine International Cohorts. *J Clin Oncol* 2019; **37:** 2632–2642.
7. Papaemmanuil E, Gerstung M, Bullinger L, Gaidzik VI, Paschka P, Roberts ND *et al.* Genomic Classification and Prognosis in Acute Myeloid Leukemia. *N Engl J Med* 2016; **374**: 2209–2221.
8. Blanche P, Dartigues J-F, Jacqmin-Gadda H. Estimating and comparing time-dependent areas under receiver operating characteristic curves for censored event times with competing risks. *Stat Med* 2013; **32**: 5381–5397.
